# Key role of oxidizing species driving water oxidation revealed by time-resolved optical and X-ray spectroscopies

**DOI:** 10.1038/s41563-026-02514-9

**Published:** 2026-02-26

**Authors:** Caiwu Liang, Lucas Garcia Verga, Benjamin Moss, Santosh Kumar, Soren B. Scott, Mark A. Turner, Pilar Ferrer, Veronica Celorrio, Dave C. Grinter, Yemin Tao, Sid Halder, Yifeng Wang, Cindy Tseng, Guangmeimei Yang, Georg Held, Sarah J. Haigh, Aron Walsh, Ifan E. L. Stephens, James R. Durrant, Reshma R. Rao

**Affiliations:** 1 Department of Materials, Imperial College London, London, UK; 2 Department of Chemistry, Centre for Processable Electronics, Imperial College London (White City Campus), London, UK; 3 Diamond Light Source, Harwell Science and Innovation Campus, Didcot, UK; 4 Department of Chemistry, University of Copenhagen, Copenhagen, Denmark; 5 Department of Materials, University of Manchester, Manchester, UK; 6 Department of Chemistry, Chemistry Research Laboratory, Oxford University, Oxford, UK; 7 Present Address: Division of Chemistry and Chemical Engineering, California Institute of Technology, Pasadena, CA USA

**Keywords:** Electrocatalysis, Electrocatalysis, Catalytic mechanisms

## Abstract

Oxidation states underpin the understanding of active states, reaction mechanisms and catalytic performance of electrocatalysts. However, determining them at complex solid–liquid interfaces is challenging. Here we use multimodal spectroscopy to investigate polarized iridium oxide (IrO_*x*_) electrodes, a model water oxidation catalyst, to identify potential-dependent iridium and oxygen oxidation states. By integrating multiple operando spectroscopies (optical (ultraviolet–visible), Ir L-edge and O K-edge X-ray absorption spectroscopy) with electrochemistry mass spectrometry and density functional theory calculations, we identify the sequential depletion of electron densities from the Ir5*d* band (corresponding to Ir^3+^→Ir^4+^→Ir^5+^), followed by electron removal from the O2*p* band, forming electrophilic oxygen species (O^−1^) due to enhanced Ir–O covalency and electronic state overlap. Time-resolved measurements reveal distinct lifetimes for Ir^5+^ and O^−1^ states under water oxidation conditions, Ir^5+^ remains unreactive whereas O^−1^ is consumed at a time constant commensurate with the reaction rate, indicating that O^−1^ drives the oxygen evolution reaction. These findings demonstrate the necessity of using multiple operando techniques to gain a unified understanding of the evolution of oxidation states and active sites with potential for water oxidation on oxide catalysts.

## Abstract (web summary)

Iridium oxide is a state-of-the-art catalyst for green hydrogen production, but the identity of species present under operating conditions remains debated. Here, using multiple operando spectroscopy approaches with time-resolution, water oxidation is found to be driven by O^−1^ states.

## Main

The oxidation state of an element is defined as the net charge an atom would have if all the bonding electrons in heteronuclear bonds were assigned to the more electronegative atom^1^. It provides a straightforward way to understand and predict chemical bonding, redox reactions and materials properties. In practice, atoms in solids often share their electron densities because bonding is seldom purely ionic^2,3^; it is generally heteropolar, exhibiting varying degrees of covalency and ionicity. Consequently, connecting oxidation states to the observable electron density distribution in solid-state materials is often ambiguous.

In electrochemical systems, which inherently involve redox processes, assigning oxidation states and interpreting atomic charge can be complicated^4,5^. In lithium batteries, for instance, the traditional view of transition-metal-centred redox processes for Li metal oxide cathodes has expanded to include oxygen redox processes within the oxide lattice, where oxygen may deviate from its conventional –2 oxide state and exist in a –1 state. However, whether this oxygen –1 state arises from localized O–O dimers, peroxo-like species or if it involves (de)localized holes on oxygen atoms within the metal oxide, remains under debate^6–9^.

The complexities of determining oxidation states in electrocatalysts are even more pronounced. Unlike battery materials, where redox processes typically occur within the bulk, electrocatalytic reactions take place at the surface, where the local stoichiometry and electronic structure^10^ deviate from the bulk^11–13^. The challenge of experimentally measuring surfaces at solid–liquid interfaces further complicates the assignment of active sites and species present^14^. Surface Pourbaix diagrams from density functional theory (DFT) calculations have enabled substantial progress in understanding the evolution of surface adsorbates; however, they do not provide a detailed picture of how charges are distributed between the metal and ligand sites^15–17^.

Iridium oxides (IrO_*x*_) have been extensively studied as water oxidation catalysts, since they exhibit both high activity^18^ and stability^19,20^ and serve as a model system for understanding solid–liquid interfaces^21–23^, yet debate persists about the oxidation states under operating conditions and, more importantly, the active states that drive water oxidation. Operando X-ray absorption spectroscopy (XAS) and X-ray photoelectron spectroscopy studies have suggested the formation of Ir^4+^ (ref. ^24^), Ir^4.*x*+^ (ref. ^25^) or Ir^5+^ species at oxygen evolution reaction (OER) potentials^26–28^, whereas other reports propose Ir^6+^ (refs. ^29,30^). By contrast, recent near-edge X-ray absorption fine structure (NEXAFS) studies propose the formation of electrophilic oxygen species as key drivers for water oxidation^31–34^. The assignment of electron-deficient oxygen is primarily based on a pre-edge feature, observed at ~529 eV in the O K-edge spectra of iridium oxides. However, Klingenhof et al. assigned the ~529-eV signal to O ligands in the bulk after the α-to-γ transformation on NiX (X = Fe, Mn or Co) layer double hydroxide catalysts^35^. Similarly, ref. ^36^ linked the 528–529-eV feature in H_3.6_ IrO_4_·3.7H_2_O to structural water, noting that removing water diminished the signal. For reference, liquid water absorbs at higher energies with the pre edge at ~535 eV (refs. ^37,38^). A recent report suggested that the ~529-eV signal could be due to highly oxidized states of iridium, which shifts the absorption of oxygen due to the covalent Ir–O bond^39^. Despite extensive operando studies, there remains no consensus on whether the key reactive species in IrO_*x*_ are metal centred (Ir^n+^) or oxygen ligand centred (O-based holes), representing a long-standing mechanistic ambiguity in the field. This challenge arises from the inherent ambiguity of charge distribution during electrochemical oxidation processes, and is compounded by the fact that each spectroscopic technique offers only partial information, whereas a molecular view of the interface demands the combination of multiple operando techniques with a sufficient time resolution.

Here we combine multiple operando spectroscopies, mass spectrometry and theoretical methods to map the oxidation states of IrO_*x*_ and to identify and quantify the active oxidizing species that drive water oxidation. First, we use quantum mechanical calculations to probe the electron density distribution on surface atoms under varying potentials, linking these distributions to possible oxidation state changes. We then integrate operando optical spectroscopy (based on our recent work^22^) with operando XAS at the Ir L edge and O K edge to track the oxidation state evolution of both metal centres and oxygen ligands. Time-resolved X-ray and optical spectroscopy are used to capture the dynamics of these states, illuminating their roles in the dynamic catalytic cycle. Using this multimodal, time-resolved approach, we demonstrate that surface oxidation at low potentials is dominated by the metal 5*d* states, whereas that at high potentials is dominated by the O2*p* states, leading to the formation of electrophilic O^−^^1^ species. This species exhibits a time constant consistent with the overall reaction rate, suggesting its role as the key oxidizing species driving OER. These insights provide a detailed molecular view of the water oxidation interface, and demonstrate the importance of the covalent nature of metal oxide bonds in the formation of the active species for water oxidation.

## Potential-dependent surface oxidation and localized charge density

**Fig. 1:**
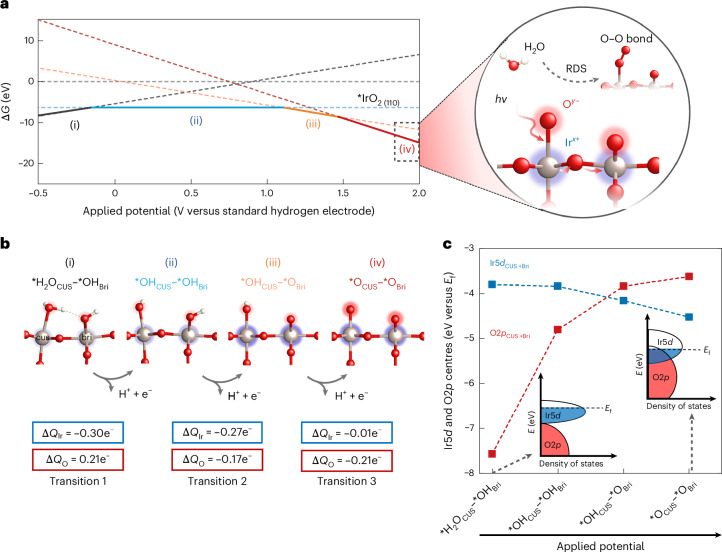
Evolution of surface intermediates, electronic structure and charge redistribution with potential on IrO_2_ surfaces. **a**, Potential-dependent coverage of adsorbates on a modelled IrO_2_ structure from DFT calculations (left) and schematic showing the calculated structure before the rate-determining step (RDS) of water oxidation and O–O bond formation, highlighting the ambiguous oxidation states and charge distribution for this critical intermediate (right). **b**, Structures involved in different redox transitions from DFT calculations and the corresponding sum of average change in Bader charges (Δ*Q*_Ir_ and Δ*Q*_O_) for both CUS and bridge Ir and O atoms present at IrO_2_ surfaces during each redox reaction. Positive (negative) numbers indicate an increase (decrease) in the number of electrons (Supplementary Note 1 shows the detailed calculations). The blue and red shading surrounding the iridium and oxygen atoms are simple guides for the eye to indicate positive charge and negative charge, respectively, with the size and intensity of the shading reflecting the magnitude of charge change. **c**, Average Ir5*d* and O2*p* band centre energies of the occupied states (versus the Fermi level *E*_f_) of surface atoms for different surface adsorbate structures (Supplementary Fig. 2 shows the separated band centres of CUS and Bri sites). The inset scheme illustrates the overlapping of Ir5*d* with O2*p* bands as the surface structure evolves with increasing applied potential and the upshift of O2*p* bands (Supplementary Note 1 shows the projected density of states). Source data

On increasing the potential, changes in the surface oxygenated species can be predicted by surface Pourbaix diagrams (Fig. 1a and Supplementary Table 1), calculated using DFT. This result is consistent with previously established surface Pourbaix diagrams^15,16,22,40–44^, and is not strongly influenced by the Hubbard *U* corrections for Ir*d* states (Supplementary Fig. 1). The changes in surface structures, related to redox transitions (also reported in our recent work^22^) are (Fig. 1b and Supplementary Note 1)

1 $${\mathrm{Redox}}\,{\mathrm{transition}}\,1:{\ast\atop}{{\rm{H}}}_{2}{\rm{O}}_{\mathrm{CUS}}+{\ast\atop}{{\rm{H}}}_{\mathrm{Bri}}\to {\ast\atop }{\mathrm{OH}}_{\mathrm{CUS}}+{\ast\atop }{{\rm{H}}}_{\mathrm{Bri}}+{{\rm{H}}}^{+}+{{\rm{e}}}^{-},$$

2 $${\mathrm{Redox}}\,{\mathrm{transition}}\,2:{\ast\atop }{\mathrm{OH}}_{\mathrm{CUS}}+{\ast\atop }{{\rm{H}}}_{\mathrm{Bri}}\to {\ast\atop }{\mathrm{OH}}_{\mathrm{CUS}}+{\ast\atop }{\mathrm{Bri}}+{{\rm{H}}}^{+}+{{\rm{e}}}^{-},$$

3 $${\mathrm{Redox}}\,{\mathrm{transition}}\,3:{\ast\atop }{\mathrm{OH}}_{\mathrm{CUS}}\to {\ast\atop }{{\rm{O}}}_{\mathrm{CUS}}+{{\rm{H}}}^{+}+{{\rm{e}}}^{-}.$$

CUS refers to coordinatively unsaturated Ir sites and Bri denotes bridge O sites bonded to two adjacent Ir atoms. To investigate the possible changes in charge density associated with these surface structural transitions, we calculated the average changes in Bader charges for surface Ir and O atoms during each redox transition (Fig. 1b, Supplementary Table 2 and Supplementary Fig. 2). Bader charges are obtained by dividing the electron density into atomic regions using surfaces at which the density gradient is zero. The results indicate substantial electron depletion predominantly occurring at Ir sites for redox transitions 1 and 2 (charge decreases of approximately –0.3e^−^ and –0.27e^−^, respectively). Conversely, at higher potentials (redox transition 3), electron depletion is primarily observed on O atoms. Similar trends were observed for the (100) surface of hollandite IrO_2_, representative of a more open structure resembling amorphous IrO_*x*_ (refs. ^22,45^) (Supplementary Fig. 3). Furthermore, we calculated the evolution of the average Ir5*d* and O2*p* band centres of occupied states for surface atoms (Fig. 1c). As the potential increases, the O2*p* band centre shifts upward from approximately –7.6 eV to around –3.8 eV, whereas the Ir5*d* band centre shifts downward from approximately –3.9 eV to around –4.5 eV due to small changes in the Ir5*d* band occupation near the Fermi level and changes in the overall shape of the Ir5*d* band (Supplementary Fig. 4). Therefore, at lower potentials, oxidation depletes the electron density from the Ir5*d* bands. As the potential increases, both Ir5*d* and O2*p* bands are close to the Fermi level, thereby initiating electron extraction from the O2*p* bands (Fig. 1c, inset). These theoretical results suggest that oxidation on IrO_*x*_ may involve both Ir and O sites, and the dual-site participation in electron loss could complicate the assignment of oxidation states and the mechanistic understanding of water oxidation (Fig. 1a).

## Determination of Ir and O oxidation using correlated spectroscopies

**Fig. 2:**
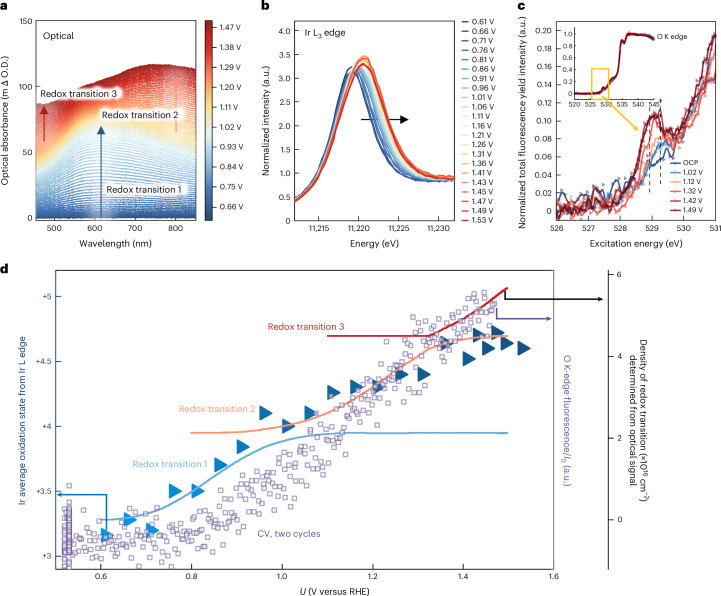
Correlating optical, hard and soft X-ray spectroscopies to identify Ir and O oxidation processes. **a**, Difference ultraviolet–visible absorbance spectra of IrO_*x*_ during a linear sweep scan from 0.66 V_RHE_ to 1.48 V_RHE_ in 0.1-M HClO_4_ at a scan rate of 1 mV s^−1^ (iR corrected). The absorption changes are calculated with respect to the absorption at 0.66 V_RHE_. Absorption changes were recorded after five cyclic voltammetry cycles, at every 1 mV and shown at every 5 mV. Arrows indicate the dominant wavelengths at which the absorbance increases with potential, representing three different redox transitions. **b**, Ir L_3_-edge XANES region of electrodeposited IrO_*x*_ on FTO substrate measured at different potentials in 0.1-M HClO_4_. Data adapted from our previous work^21^. **c**, Insets: steady-state operando NEXAFS O K-edge spectra of amorphous IrO_*x*_ deposited on gold-coated SiN_*x*_ substrates measured at various applied potentials in 0.1-M HClO_4_ electrolyte. Potential values are given versus a reversible hydrogen electrode (RHE). The data are normalized to the intensity at around 540 eV and calibrated using the pre edge of water at 535 eV (refs. ^37,38^). The main figure shows a zoomed-in view with energy ranging from 526 to 531 eV. Dashed lines show two peak positions at an increase of around 528.9 eV and 529.3 eV with the applied potentials. **d**, Comparison between the iridium oxidation state determined from the white-line position in **b** (left *y* axis), the sum of deconvoluted densities of redox transitions 1, 2 and 3 from optical spectroscopy (black, right *y* axis) and the fluorescence intensity at 529 eV in the O K edge over two cyclic voltammetry cycles. The *y* axis scales are adjusted for visual clarity. Source data

With theoretical evidence demonstrating that Ir and O could both be oxidized, we use a combination of operando optical (Fig. 2a), Ir L-edge (Fig. 2b) and O K-edge (Fig. 2c) spectroscopies to experimentally capture the changes in oxidation states. Electrodeposited IrO_*x*_ thin films are used considering they are volume active, that is, a majority of the Ir centres are active^22,46^. Transmission electron microscopy analysis (Supplementary Note 2 and Supplementary Fig. 5) shows no evidence of crystallinity in these films, indicating that the material is composed of short-range ordered [IrO_6_] octahedra^22^. We note that this IrO_*x*_ is highly porous, with channels for electrolyte, in contrast to the long-range ordered rutile IrO_2_, which exhibits a more well-defined interface. Nevertheless, our previous optical spectroscopy studies have shown that these materials share similar redox chemistry, although amorphous IrO_*x*_ exhibits a much higher density of accessible sites for redox transitions and OER^22^. Recent studies have also shown comparable changes in Ir4*f* X-ray photoelectron spectroscopy as well as the O K-edge and Ir L_3_-edge XAS spectra for amorphous and crystalline iridium oxides^26,47^, suggesting that the observation on amorphous IrO_*x*_ could reasonably extend to rutile systems.

Operando optical spectroscopy shows an increase in absorbance with potential (Fig. 2a). Following our previous work, these can be deconvoluted into three different redox transitions according to their spectral shape, and their densities are quantified using the Lambert–Beer law (light blue, orange and red lines in Fig. 2d, black *y* axis; Supplementary Note 3 and Supplementary Figs. 6–8 provide the deconvolution details)^21,22^. Similarly, operando Ir L-edge XANES shows that the white-line position continuously shifts to a higher energy with increasing potential from 0.6 V to ~1.4 V_RHE_ (Fig. 2b, adapted from our previous work^21^), but remains roughly unchanged at higher potentials. This energy shift corresponds to an increase in the average iridium oxidation state from approximately +3.1 to around +4.7 (Fig. 2d, blue triangles). Interestingly, the potential dependence of the first two redox transitions detected via optical spectroscopy correlate well with the increase in Ir oxidation state (that is, the light blue lines and orange line overlap well with the blue triangles), thereby corresponding to the oxidation of Ir^3+^ to Ir^4+^ and then Ir^4+^ to Ir^5+^. Although the average Ir oxidation state is +4.7, the redox active sites are probably Ir^5+^ with a minor fraction of Ir^3+^ and Ir^4+^. This is consistent with recent reports showing that IrO_*x*_ attains a surface oxidation state of Ir^5+^ at OER-relevant potentials^26^. Crucially, the final redox transition observed in optical spectroscopy, occurring in the OER region, does not correspond to further oxidation of the Ir centre. Instead, as the Ir oxidation state plateaus, redox transition 3 emerges (Fig. 2d, red lines). This observation suggests that there is an additional oxidation process in which electrons are extracted without further oxidizing Ir. We note that the above-mentioned spectroscopic changes are reversible with potential, and no substantial changes are observed in the electrochemistry (Supplementary Fig. 9), crystallinity (Supplementary Figs. 10 and 11), surface composition (Supplementary Fig. 12) or optical spectroscopy measurements after 20 cycles (Supplementary Fig. 13; Supplementary Note 4 provides a detailed discussion).

To further understand the physicochemical nature of the final redox transition, operando NEXAFS was used to examine changes in the oxygen ligands (Supplementary Note 5 and Supplementary Fig. 14). The operando O K-edge spectra resemble those of pure water (Fig. 2c, inset)^37,38^ but display distinct features in the low-energy region from 527 eV to 532 eV associated with IrO_*x*_ (Fig. 2c and Supplementary Figs. 15 and 16). With increasing potential from the open-circuit potential (OCP) to 1.49 V_RHE_, there is an increase in intensity between 528.5 and 530 eV. This observation aligns with findings from refs. ^31–34^ on IrO_*x*_ and refs. ^35,48,49^ on Ni-Fe based catalysts. It is worth noting that assigning O K-edge features is not straightforward, as the absorption energies of oxygen species can vary across metal oxides^32,34,36,39,50^. Considering this challenge, rather than assigning specific peaks to different species, we traced the signal at 529 eV with potential during two cyclic voltammetry cycles from 0.5 V_RHE_ to ~1.47 V_RHE_ (Fig. 2d, purple dots), and correlated it with the Ir oxidation states and optically resolved redox transitions (Supplementary Fig. 17). The oxygen XAS signal remains constant in the low-potential range (0.5–0.85 V_RHE_), but increases continuously at a higher potential up to 1.47 V_RHE_. In particular, the increase in XAS signal to >1.40 V_RHE_ aligns well with redox transition 3 resolved in optical spectroscopy.

The above correlated spectroscopic results suggest that the electrons extracted during the final redox transition might come from oxygen ligands, forming electron-deficient oxygen species O^−1^. In addition, the increase in the oxygen XAS signal during redox transition 2 is attributed to changes in the signal of different oxygenated species (that is, *OH_Bri_ → *O_Bri_ + H^+ ^+ e^−^). The oxygen involved in redox transition 2 probably remains in a chemical state close to typical O^−2^, as the observed charge extraction is primarily accounted for by changes in the Ir oxidation states. Thus, by comparing Ir and O signals with optical spectroscopy, our results seem to support the existence of O^−1^ at positive potentials. However, steady-state XAS measurements have limited dynamic and potential resolution, and are susceptible to beam damage or time-dependent changes during prolonged exposure^9^, which may introduce uncertainties. Therefore, we next traced the dynamic responses of Ir and O to applied potential using time-resolved optical and X-ray spectroscopies.

## Dynamics of iridium and oxygen oxidation

**Fig. 3:**
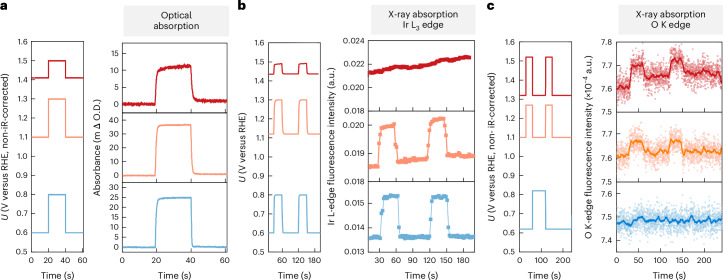
Dynamics of Ir and O oxidation probed by time-resolved operando optical and X-ray spectroscopies. **a**, Time-resolved optical absorption changes during potential step experiments on IrO_*x*_ in 0.1-M HClO_4_ electrolyte. Potentials (non-iR-corrected) were stepped from 0.6 to 0.8 V_RHE_, 1.1 to 1.3 V_RHE_ and 1.41 to 1.5 V_RHE_. Optical absorption was monitored at wavelengths of 600 nm, 800 nm and 500 nm, corresponding to the maximum absorption wavelength for redox transitions 1, 2 and 3, respectively (Supplementary Fig. 7). **b**, Changes in fluorescence intensity at the Ir L_3_ edge (11,222 eV) during potential step experiments on IrO_*x*_ deposited on an FTO substrate in 0.1-M HClO_4_ electrolyte. Potential steps were applied between 0.6 and 0.8 V_RHE_, 1.12 and 1.3 V_RHE_ and 1.44 and 1.5 V_RHE_, corresponding to redox transitions 1, 2 and 3, respectively, as identified from the spectral deconvolution in optical spectroscopy. **c**, Changes in the fluorescence intensity at the O K edge during potential step experiments in 0.1-M HClO_4_ electrolyte. Potentials (non-iR-corrected) were stepped in the ranges of 0.62–0.82 V_RHE_, 1.1–1.27 V_RHE_ and 1.32–1.52 V_RHE_. Fluorescence intensity changes were monitored at 529 eV for the potential ranges of 0.62–0.82 V_RHE_ and 1.1–1.27 V_RHE_, and at 528.7 eV for the range of 1.32–1.52 V_RHE_. Source data

The dynamic responses of the optical, Ir and oxygen XAS signals are tracked as the potential steps up and down in different potential ranges. The ranges 0.6–0.8 V_RHE_, 1.1–1.3 V_RHE_ and 1.4–1.5 V_RHE_ were selected for redox transitions 1, 2 and 3, respectively (Fig. 2d). As shown in Fig. 3a, for all three redox transitions, the optical absorption signal responds rapidly to the potential change. Similarly, the Ir XAS intensity at 11,222 eV rises and falls with the stepping up and down of potential in the ranges corresponding to redox transitions 1 and 2 (Fig. 3b).

The Ir XAS intensity at 11,222 eV is proportional to its oxidation states changes, as established by correlating the intensity with the oxidation states obtained in Fig. 2d (Supplementary Note 6 and Supplementary Figs. 18 and 19). By contrast, during redox transition 3 (1.44–1.5 V_RHE_), the Ir XAS signal does not respond to potential changes; instead, a very slow and gradual increase in the oxidation state over time was observed (Fig. 3b, top). This difference between the time-resolved optical and Ir L_3_-edge XAS spectroscopies suggests that although redox transitions 1 and 2 are associated with the oxidation of iridium, the third transition is not.

The observed slow increase in fluorescence intensity over time could indicate the time-dependent oxidation of a small amount of bulk iridium, inaccessible within shorter experimental time frames. Similar measurements on the dynamics of oxygen species reveal that the fluorescence intensity at 529 eV does not change from 0.62 to 0.82 V_RHE_, but changes with potential steps in the ranges of 1.1–1.27 V_RHE_ and 1.32–1.52 V_RHE_ (Fig. 3c). This behaviour aligns with the changes observed during cyclic voltammetry cycles (Fig. 2c). Further investigations establish that the observed changes are indeed sensitive to the selected X-ray beam energy (Supplementary Fig. 20), confirming that the changes are related to alterations in the oxygen species.

On the basis of experimental and DFT findings, the first redox transition is attributed to the Ir^3+^ to Ir^4+^ transition and a deprotonation of water into *OH on the Ir CUS site. This is consistent with recent findings suggesting that *OH on the CUS Ir site is hardly visible in the O K-edge NEXAFS^47^. The second redox transition can be associated with further oxidation from Ir^4+^ towards Ir^5+^, accompanied by the deprotonation of *OH on the bridge site into *O. This *OH to *O transformation was captured by the absorption peak at around 529.3 eV in O K-edge NEXAFS spectra^23,27,31–34,47–49^. The final redox transition, occurring in the OER-relevant potential region, involves further oxidation and the deprotonation of *OH at the CUS site to form *O^−1^, whereas Ir remains as Ir^5+^. This is consistent with our DFT calculations showing that electron depletion in this step is dominated by the oxygen ligand. This assignment differs from oxygen redox that emphasizes lattice oxygen involvement, such as anion redox in battery cathodes and lattice oxygen evolution mechanism in OER^51^. In addition, our results do not support the accumulation of a substantial coverage of higher oxidation state Ir^6+^ species in these materials, consistent with a recent report showing gas-phase IrO_3_ (Ir^6+^) only occurs as a degradation side product in rutile IrO_2_ (ref. ^52^).

## Kinetics of O^−1^ species for water oxidation

**Fig. 4:**
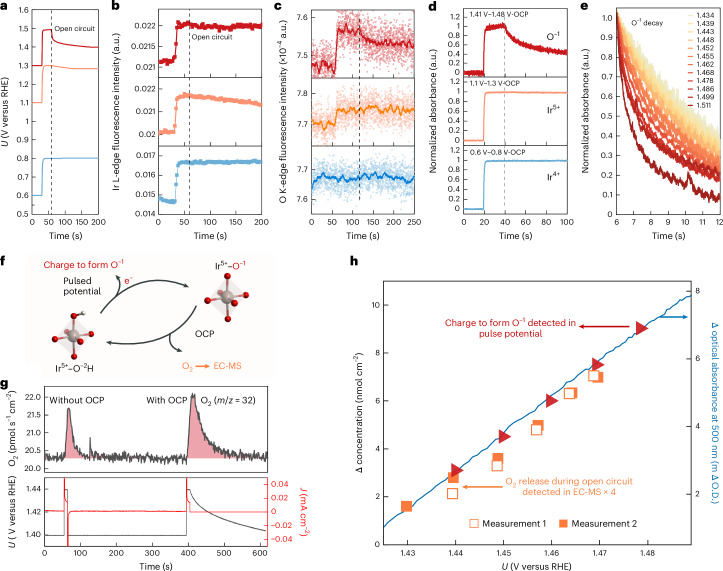
Operando tracking of O^−1^ and their decay to molecular oxygen during OCP. **a**, Potential profile of OCP decay measurement between 0.6 and 0.8 V_RHE_, 1.12 and 1.3 V_RHE_, and 1.3 and 1.5 V_RHE_ in 0.1-M HClO_4_ electrolyte. **b**, Change in fluorescence intensity of Ir L edge (11,222 eV). **c**, Change in fluorescence intensity of O K edge during OCP decay measurement between 0.62 and 0.82 V_RHE_ and 1.1 and 1.27 V_RHE_ at 529 eV, and 1.27 and 1.52 V_RHE_ at 528.7 eV. **d**, Normalized optical signal decay. The optical signals are taken at the maximum absorption wavelength for each redox transition, with redox transition forming Ir^4+^ at 600 nm, Ir^5+^ at 800 nm and O^−1^ at 500 nm (Fig. 2a and Supplementary Note 3 show the spectra). **e**, Normalized optical signal decay at 500 nm at different applied potentials. The potentials are iR corrected and in the RHE scale. **f**, Schematic showing the formation of O^−1^ on removing an electron and proton from a protonated IrO_6_ structure by raising the potentials and its corresponding decay during OCP to form molecular oxygen. **g**, O_2_ (*m*/*z* = 32) detected in EC-MS for pulsed potential measurements from 1.40 to 1.44 V_RHE_ and for a subsequent OCP decay measurement with the same pulsed potential steps and holding time. **h**, Quantitative comparison between the charge associated with O^−1^ formation and the detected molecular oxygen during OCP decay. The amount of O^−1^ formed is quantified by integrating the cathodic current peak during pulsed potential measurements, corresponding to the reduction of accumulated O^−1^ back to O^−2^ at 1.415 V_RHE_, assuming a one-to-one correspondence between O^−1^ formation and the number of electrons transferred. Above this potential, the charge is dominated by oxygen redox with negligible contribution from iridium (Fig. 2d), and double-layer charging/discharging is minor (Supplementary Note 7 and Supplementary Fig. 28). The amount of O_2_ released during OCP decay is directly measured by EC-MS, with contributions from potential-jumping controls subtracted (Supplementary Figs. 24 and 27). The change in optical absorption relative to 1.415 V_RHE_ at 500 nm (right axis) is co-plotted to correlate O^−1^ accumulation with the corresponding optical signal changes resulting from redox transitions. Source data

Although we have established that oxidizing species Ir^5+^ and O^−1^ co-exist at high potentials, it remains unclear whether Ir^5+^, O^−1^ or a cooperative interaction between them controls the rate‑determining O–O-bond‑forming step. To investigate the roles of these species in catalysing water oxidation, we determined their decay time constants by coupling the OCP decay measurements with time‑resolved optical and X‑ray spectroscopies. In this method, the potential is stepped up and held before switching to an open circuit, allowing the electrode to relax (Fig. 4a). Figure 4b shows that across all three potential regions examined, the XAS intensity of Ir remains stable after switching to the open circuit, indicating that the accumulated Ir^4+^ and Ir^5+^ species are stable. By contrast, the oxygen XAS signals at higher potentials (1.32 to 1.52 V_RHE_)—assigned to O^−1^ species—exhibit a notable decay, suggesting the consumption of these oxygen species during open-circuit conditions (Fig. 4c). These dynamic behaviours of iridium and oxygen species were also clearly observed using time-resolved optical spectroscopy (Fig. 4d). We note that the loss of spectral features during the initial decay closely matches those associated with redox transition 3, which generates O^−1^ species (Supplementary Fig. 21). In addition, the initial decay rate increases with the applied potential (Fig. 4e), with the time required for the initial 25% decay decreasing from approximately 7 s at 1.43 V to 0.9 s at 1.51 V_RHE_, indicating a quicker consumption of O^−1^ species at higher potentials and at higher coverages of O^−1^. Similar decay trends were observed in time-resolved O K edge absorption spectroscopy (Supplementary Fig. 22). The stark difference in the behaviour of highly oxidized Ir species and electron-deficient O^−1^ species, as evidenced by both optical and X-ray spectroscopies, suggests that Ir-oxidizing species does not change the oxidation state under OER conditions; instead, O^−1^ is dynamically changing. These results suggest that the fast kinetics of O–O bond formation and oxygen molecule release on IrO_*x*_ can be attributed to the accumulation of O^−1^ species at high potentials, resulting from the high covalency of Ir–O on oxidation (Fig. 4f), thereby supporting the notion that O^−1^ species drive water oxidation^23,28,31–34^.

To further understand the correlation between O^−1^ and oxygen evolution, we quantified oxygen release during OCP decay using highly sensitive (resolution, subpicomole per second) on-chip electrochemical mass spectrometry (EC-MS)^53^. Oxygen signals were measured using two sequential procedures: an OCP decay measurement and a step potential measurement as control (Supplementary Note 7). In particular, the OCP decay measurement shows a larger oxygen signal compared with the step potential measurement, indicating additional oxygen release during open-circuit decay (Fig. 4g). Reversing the order of the two procedures yielded consistent results (Supplementary Fig. 23). Measurements at potentials from 1.44 to 1.48 V_RHE_, conducted in two independent experiments, consistently showed increased oxygen release during OCP, which was quantified (Supplementary Note 7 and Supplementary Figs. 24–26). Interestingly, the net amount of oxygen produced during OCP decay shows an almost-linear increase with the applied potential before switching to the open circuit. A similar linear increase is observed in the 500-nm absorbance signal and the accumulation of O^−1^ species (Fig. 4h; Supplementary Fig. 27 shows the quantification of O^−1^). In particular, the O^−1^ concentration is almost four times the amount of oxygen released during OCP decay, which suggests that the release of every O_2_ from the water molecules gives rise to four electrons to discharge four accumulated O^−1^ species. These results indicate that the consumption of O^−1^ is quantitatively linked to the release of oxygen during open-circuit decay, suggesting that the O^−1^ species could be involved in the step of forming the O–O bond and subsequent oxygen release. In addition, inductively coupled plasma mass spectrometry measurements show that Ir dissolution during decay is 2–3 orders of magnitude lower than the amount of oxygen released (Supplementary Fig. 29 and Supplementary Note 8), indicating that dissolution is a minor contribution. On the basis of previous established DFT calculations for IrO_2_(110) surfaces—which suggest that the transition from *O to *OOH (that is, the O–O bond formation) is the rate-determining step^22,23,40,41^—we propose a mechanism for the open-circuit decay process, analogous to our previous work^22^. In this process, an active state (O^−1^) undergoes a nucleophilic attack by a water molecule, leading to O–O bond formation and subsequent oxygen release. To maintain charge balance without external electron transfer (as that during open-circuit conditions), other O^−1^ species in the network accept a proton and become reduced, regenerating O^−2^ in the form of *OH on the surface. Although this mechanism is specific to the OCP decay, it involves a fundamental catalytic cycle with charged surface oxygen species (O^−1^), water molecules, O–O bond formation and oxygen release. The key distinction between OCP decay and constant potential operation is the presence of an externally applied potential during the latter, which facilitates continuous electron extraction from the catalyst. This external potential enables the rapid reoxidation of reduced O^−2^ sites back to O^−1^ species, allowing them to participate in successive catalytic cycles releasing O_2_. Therefore, we consider the mechanism described for OCP decay representing a single catalytic cycle of the OER on iridium oxides.

Under anodic polarization, the progressive oxidation of Ir enhances Ir–O covalency and brings the Ir5*d* and O2*p* band centres into closer energetic alignment, thereby promoting electron depletion from surface oxygen atoms and enabling the formation of O^−1^ species necessary for driving O–O bond formation. The ability of iridium oxides to stabilize such electron-deficient oxygen states may arise from the unique covalency of surface Ir–O bonds under high potentials—a property that may be fundamental to their exceptionally fast OER kinetics. Further analysis of reaction rate versus O^−1^ concentrations suggests that IrO_*x*_ is unlikely to follow a simple rate law kinetics since we observed an unphysically high reaction order (Supplementary Fig. 30 and Supplementary Note 9); instead, our data align better with a kinetic model in which the reaction rate increases approximately exponentially with the accumulation of O^−1^ species. These results agree with previous reports, which suggest that repulsive interactions exist between oxo species and lower the activation barrier of the rate-determining step and, thus, promote water oxidation^21–23,54^. Future work should also directly probe whether the accumulation of O^−1^ species influences dissolution, opening up pathways to jointly optimize the OER activity and stability. As these reactive O^−1^ configurations are accessible only under operando conditions, our results underscore that an accurate understanding of catalytic activity should account for the electronic structure of the oxidized surface under very positive potential, rather than relying solely on bulk stoichiometric models. As a result, future research focused on tuning surface metal–oxygen covalency and band alignment at high potentials can open promising avenues for the design of more active OER catalysts.

## Conclusions

In summary, we used multimodal, time-resolved spectroscopies to elucidate the potential-dependent oxidation states of iridium and oxygen in iridium oxide and its impact on water oxidation. We found a sequential oxidation process, which first involves iridium oxidation (Ir^3+^/Ir^4+^ and Ir^4+^/Ir^5+^) at low potentials, followed by electron depletion from oxygen ligands at higher potentials, primarily due to the increased covalency of Ir–O, leading to the formation of electrophilic O^−1^ at OER potentials. Time-resolved spectroscopies show that the lifetime of oxidized iridium (Ir^5+^) is much longer than the time constant of oxygen release, whereas the kinetics of electrophilic oxygen O^−1^ align closely with OER. These suggest that electrophilic oxygen, rather than the highly oxidized iridium, plays a key role in driving the rate-determining-step O–O bond formation. EC-MS results show that the concentration of O^−1^ species is quantitatively correlated to the amount of molecular oxygen released, with the consumption of roughly four O^−1^ for the release of every oxygen molecule. These results provide a unified mechanistic picture that resolves long-standing discrepancies over whether Ir-centred or oxygen-ligand-centred species act as the reactive oxidants during water oxidation. Our results also demonstrate the necessity of multimodal, time-resolved characterization techniques to gain a holistic understanding of complex solid–liquid interfaces. Beyond OER on metal oxides, identifying potential-dependent oxidation states and their dynamics serves as an important tool to determine how charge transfer occurs during rate-determining catalytic steps for a number of reactions such as CO_2_ reduction and oxygen reduction.

## Methods

### DFT calculations

Spin-polarized DFT calculations were performed using the Vienna ab initio simulation package^55,56^ and the projector augmented-wave method^57,58^. We used the Perdew–Burke–Ernzerhof exchange–correlation functional^59^ for all the calculations with and without the inclusion of Hubbard *U* correction using the rotationally invariant DFT + *U* formalism^60^ with *U* = 2.0 eV (ref. ^61^) for the *d* states of Ir atoms. The (110) surface of an IrO_2_ rutile structure was modelled with a symmetric 3 × 1 slab with at least 30 Å of vacuum and six atomic layers, of which the two central layers were fixed in the optimized bulk geometry. Adsorbates were symmetrically placed on both sides of the slab at the Ir_Cus_ and Ir_Bri_ sites. The calculations were performed with a plane-wave energy of 500 eV and a 3 × 4 × 1 *k*-point mesh for the Brillouin zone integration. The convergence criteria were set to 10^−4^ eV for the electronic self-consistent iteration and 0.025 eV Å^−1^ for the atomic forces on all atoms during ionic relaxations. The computational hydrogen electrode was used to consider the effect of the applied potential^17^ and the free energy changes as a function of applied potential were defined as

4 $$\Delta {G}_{\mathrm{ads}}({\rm{U}})={{E}_{\mathrm{ads}}}^{\mathrm{DFT}}+\mathrm{ZPE}+\int C{\rm{d}}T-T\Delta S-neU,$$

where *n* is the number of electrons for the considered reaction, and the ZPE, ∫*C*d*T* and *T*∆*S* terms were obtained from vibrational frequencies calculated via DFT used within the thermochemistry module from the atomic simulation environment package using the harmonic limit^62^ (Supplementary Table 1).

### Synthesis of IrO_*x*_

The hydrous amorphous IrO_*x*_ is prepared using an electrodeposition method reported in our previous reports^21,22^. A solution containing 0.2 mmol of IrCl_3_ hydrate (Fluorochem) and 1 mmol of oxalic acid dehydrate (Sigma-Aldrich) in 30 ml of water was prepared. The pH was adjusted to 10 with 5 mmol of K_2_CO_3_ (Sigma-Aldrich, ≥99.0%). The volume of the solution was then increased to 50 ml by adding another 20 ml of water. The solution was left to rest for 4 days at 35 °C and then stored in the refrigerator at 4 °C. The electrodeposition of IrO_x_ was conducted in a typical three-electrode setup in this solution. A Pt foil and an Ag/AgCl electrode are used as the counter and reference electrodes, respectively. IrO_*x*_ is deposited onto conductive substrates by applying an anodic current density of 35 µA cm^−2^ for ~1,000 s. Fluorine‑doped tin oxide (FTO) glass was used as the substrate for operando optical spectroscopy and Ir L-edge XAS measurements. Au-coated Si_3_N_4_ membranes were used for operando O K-edge XAS measurements, whereas glassy carbon was used as the substrate for EC-MS experiments.

### Operando optical absorption spectroscopy

Operando optical absorption spectroscopy was performed on ~1 cm × 1 cm IrO_x_ samples on FTO substrates using a custom-built three-electrode setup, with a Pt counter electrode and a custom-built RHE. A 10-mW tungsten–halogen light source (Thorlabs SLS201L with an SLS201C collimator) illuminated the sample, and the transmitted light was collected via a 1-cm diameter liquid light guide (Edmund Optics) and directed to a spectrometer (Andor Kymera 193i) with a charge-coupled device camera (Andor iDus Du420A-BEX2-DD), cooled to –80 °C for an improved signal-to-noise ratio. Light was collimated and refocused using two 5-cm planoconvex lenses (Edmund Optics). An Autolab potentiostat controlled the potential in the potentiostatic mode, with 10-s equilibration at each step. Optical spectra were collected by averaging 30 acquisitions (~30 ms each), and current was recorded simultaneously using custom-built LabVIEW software version 2023 Q1 (National Instruments).

### Operando NEXAFS

NEXAFS measurements were performed at the B07 beamline (B branch) at Diamond Light Source. Operando measurements were conducted using an in situ electrochemical cell described previously^63^, incorporating a 100-nm-thick Si_3_N_4_ membrane window coated with 10-nm Ti and 10-nm Au (Silson). IrO_x_ was electrodeposited directly onto the Au layer. The membrane was sealed with an O-ring, and electrical contact was made via Au pins, with Pt wire and Ag/AgCl electrodes used as the counter and reference electrodes, respectively. The Ag/AgCl reference was calibrated against RHE. A 0.1-M HClO_4_ electrolyte was continuously flowed through the cell at ~10 µl s^−1^ using a syringe pump. The electrolyte flow tube and connections to the working, reference and counter electrodes are integrated through a specially designed lid (DN63 CF flange) of the chamber, enabling it to maintain a low-pressure environment. O K-edge spectra were calibrated using the water pre edge at 535 eV and normalized to the post-edge region (~540 eV for operando and ~570 eV for ex situ measurements).

### Operando Ir L-edge XAS

Operando XAS measurements were performed using a custom-built in situ electrochemical XAS cell in our previous report^21^, enabling measurements on electrodeposited IrO_x_ films on FTO under identical conditions to operando optical experiments. The cell accommodates different substrate types and sizes and operates as a standard three-electrode configuration. XAS measurements were carried out at the B18 beamline at Diamond Light Source using a Si(111) monochromator. Reference samples (Ir powder, IrCl_3_ and IrO_2_) were measured in the transmission mode, whereas the operando measurements of amorphous IrO_x_ were measured in the fluorescence mode. Energy calibration was performed using a Pt foil referenced to the Pt L_3_ edge (11,564 eV). Before XAS, electrodes were conditioned by cyclic voltammetry between 0.6 and 1.45 V_RHE_ at 10 mV s^−1^. The XAS spectra were collected during potential holds between ~0.4 and ~1.50 V_RHE_, with ten spectra acquired and averaged at each potential. Data processing was performed using Athena version 0.9.26 (ref. ^64^). Time-resolved measurements were obtained by fixing the X-ray energy and monitoring the fluorescence intensity at 100-ms resolution, synchronized with the electrochemical data via system time stamps and normalized to the incident-beam intensity to exclude the background-intensity-fluctuating effect.

### Chip-based EC-MS measurements

In situ gas analysis during electrochemical measurements was performed using a real-time on-chip EC-MS system (Spectro Inlets). The system is based on a microporous membrane chip fabricated from semiconductor-on-insulator wafers, consisting of an array of ~2.5-µm pores distributed over a 7-mm-diameter area. The electrolyte layer thickness was limited to ~100 µm to enable the rapid transfer of dissolved gases into the sampling volume for detection. IrO_x_ catalysts were electrodeposited on glassy carbon substrates and measured in a cell geometry analogous to a rotating disk electrode configuration. Mass spectrometry and electrochemical data were synchronized and analysed using the open-source package IXDAT (https://ixdat.readthedocs.io/en/latest/). Quantification of gaseous products was achieved by calibration against oxygen evolution on an oxidized Pt-disc electrode, assuming ~100% Faradaic efficiency at high potentials.

## Online content

Any methods, additional references, Nature Portfolio reporting summaries, source data, extended data, supplementary information, acknowledgements, peer review information; details of author contributions and competing interests; and statements of data and code availability are available at 10.1038/s41563-026-02514-9.

## Supplementary information

Supplementary Information Supplementary Figs. 1–30, Notes 1–9 and Tables 1 and 2.

## Source data

Source Data Fig. 1 Surface adsorbates and band centre energy from DFT calculation data plotted in Fig. 1a,b.

Source Data Fig. 2 Correlation between optical (ultraviolet–visible), Ir L-edge and O K-edge XAS data plotted in Fig. 2a–d.

Source Data Fig. 3 Time-resolved optical, Ir L-edge and O K-edge XAS data plotted in Fig. 3a–c.

Source Data Fig. 4 Open-circuit decay of oxidizing species and EC-MS data plotted in Fig. 4a–e,g,h.

## Data Availability

The data supporting the findings of this study are available in the Supplementary Information. The datasets and analysis scripts underlying the figures are available via Zenodo at 10.5281/zenodo.18320343 (ref. ^65^). Source data are provided with this paper.

## References

[1] McNaught, A. D. & Wilkinson, A. *Compendium of Chemical Terminology* (Blackwell Science, 1997).

[2] Walsh, A., Sokol, A. A., Buckeridge, J., Scanlon, D. O. & Catlow, C. R. A. Oxidation states and ionicity. *Nat. Mater.* **17**, 958–964 (2018). 30275565 10.1038/s41563-018-0165-7

[3] Walsh, A., Sokol, A. A., Buckeridge, J., Scanlon, D. O. & Catlow, C. R. A. Electron counting in solids: oxidation states, partial charges, and ionicity. *J. Phys. Chem. Lett.* **8**, 2074–2075 (2017). 28468501 10.1021/acs.jpclett.7b00809

[4] Kuznetsov, D. A. et al. Tuning redox transitions via inductive effect in metal oxides and complexes, and implications in oxygen electrocatalysis. *Joule* **2**, 225–244 (2018).

[5] Luo, K. et al. Charge-compensation in 3*d*-transition-metal-oxide intercalation cathodes through the generation of localized electron holes on oxygen. *Nat. Chem.* **8**, 684–691 (2016). 27325095 10.1038/nchem.2471

[6] Marie, J.-J. et al. Trapped O_2_ and the origin of voltage fade in layered Li-rich cathodes. *Nat. Mater.* **23**, 818–825 (2024). 38429520 10.1038/s41563-024-01833-z PMC11150160

[7] Sathiya, M. et al. Reversible anionic redox chemistry in high-capacity layered-oxide electrodes. *Nat. Mater.* **12**, 827–835 (2013). 23852398 10.1038/nmat3699

[8] Assat, G. & Tarascon, J.-M. Fundamental understanding and practical challenges of anionic redox activity in Li-ion batteries. *Nat. Energy* **3**, 373–386 (2018).

[9] Gao, X. et al. Clarifying the origin of molecular O_2_ in cathode oxides. *Nat. Mater.* **24**, 743–752 (2025). 40065092 10.1038/s41563-025-02144-7

[10] Lunger, J. R. et al. Towards atom-level understanding of metal oxide catalysts for the oxygen evolution reaction with machine learning. *npj Comput. Mater.* **10**, 80 (2024).

[11] Haase, F. T. et al. Role of Fe decoration on the oxygen evolving state of Co_3_O_4_ nanocatalysts. *Energy Environ. Sci.* **17**, 2046–2058 (2024). 38449571 10.1039/d3ee02809g PMC10913145

[12] Hubert, M. A. et al. Characterization of a dynamic Y_2_Ir_2_O_7_ catalyst during the oxygen evolution reaction in acid. *J. Phys. Chem. C* **126**, 1751–1760 (2022).

[13] Yang, F. et al. Dynamics of bulk and surface oxide evolution in copper foams for electrochemical CO_2_ reduction. *Commun. Chem.* **7**, 66 (2024). 38548895 10.1038/s42004-024-01151-0 PMC10978924

[14] Suntivich, J. et al. Probing intermediate configurations of oxygen evolution catalysis across the light spectrum. *Nat. Energy* **9**, 1191–1198 (2024).

[15] Rossmeisl, J., Qu, Z. W., Zhu, H., Kroes, G. J. & Nørskov, J. K. Electrolysis of water on oxide surfaces. *J. Electroanal. Chem.* **607**, 83–89 (2007).

[16] Man, I. C. et al. Universality in oxygen evolution electrocatalysis on oxide surfaces. *ChemCatChem* **3**, 1159–1165 (2011).

[17] Nørskov, J. K. et al. Origin of the overpotential for oxygen reduction at a fuel-cell cathode. *J. Phys. Chem. B* **108**, 17886–17892 (2004). 39682080 10.1021/jp047349j

[18] McCrory, C. C. L., Jung, S., Peters, J. C. & Jaramillo, T. F. Benchmarking heterogeneous electrocatalysts for the oxygen evolution reaction. *J. Am. Chem. Soc.* **135**, 16977–16987 (2013). 24171402 10.1021/ja407115p

[19] Geiger, S. et al. The stability number as a metric for electrocatalyst stability benchmarking. *Nat. Catal.* **1**, 508–515 (2018).

[20] Cherevko, S. et al. Oxygen and hydrogen evolution reactions on Ru, RuO_2_, Ir, and IrO_2_ thin film electrodes in acidic and alkaline electrolytes: a comparative study on activity and stability. *Catal. Today* **262**, 170–180 (2016).

[21] Liang, C. et al. Role of electrolyte pH on water oxidation for iridium oxides. *J. Am. Chem. Soc.* **146**, 8928–8938 (2024). 38526298 10.1021/jacs.3c12011 PMC10996014

[22] Liang, C. et al. Unravelling the effects of active site density and energetics on the water oxidation activity of iridium oxides. *Nat. Catal.* **7**, 763–775 (2024).

[23] Nong, H. N. et al. Key role of chemistry versus bias in electrocatalytic oxygen evolution. *Nature* **587**, 408–413 (2020). 33208960 10.1038/s41586-020-2908-2

[24] Saveleva, V. A. et al. Operando evidence for a universal oxygen evolution mechanism on thermal and electrochemical iridium oxides. *J. Phys. Chem. Lett.* **9**, 3154–3160 (2018). 29775319 10.1021/acs.jpclett.8b00810

[25] Hillman, A. R., Skopek, M. A. & Gurman, S. J. X-ray spectroscopy of electrochemically deposited iridium oxide films: detection of multiple sites through structural disorder. *Phys. Chem. Chem. Phys.* **13**, 5252–5263 (2011). 21173969 10.1039/c0cp01472a

[26] Diklić, N. et al. Surface Ir^+5^ formation as a universal prerequisite for O_2_ evolution on Ir oxides. *ACS Catal.* **13**, 11069–11079 (2023).

[27] Nong, H. N. et al. A unique oxygen ligand environment facilitates water oxidation in hole-doped IrNiO_*x*_ core–shell electrocatalysts. *Nat. Catal.* **1**, 841–851 (2018).

[28] Velasco-Vélez, J.-J. et al. Surface electron-hole rich species active in the electrocatalytic water oxidation. *J. Am. Chem. Soc.* **143**, 12524–12534 (2021). 34355571 10.1021/jacs.1c01655 PMC8397309

[29] Li, A. et al. Atomically dispersed hexavalent iridium oxide from MnO_2_ reduction for oxygen evolution catalysis. *Science* **384**, 666–670 (2024). 10.1126/science.adg5193 38723092

[30] Retuerto, M. et al. Highly active and stable OER electrocatalysts derived from Sr_2_MIrO_6_ for proton exchange membrane water electrolyzers. *Nat. Commun.* **13**, 7935 (2022). 36566246 10.1038/s41467-022-35631-5 PMC9789951

[31] Pfeifer, V. et al. The electronic structure of iridium and its oxides. *Surf. Interface Anal.* **48**, 261–273 (2016).

[32] Pfeifer, V. et al. The electronic structure of iridium oxide electrodes active in water splitting. *Phys. Chem. Chem. Phys.* **18**, 2292–2296 (2016). 26700139 10.1039/c5cp06997a

[33] Pfeifer, V. et al. Reactive oxygen species in iridium-based OER catalysts. *Chem. Sci.* **7**, 6791–6795 (2016). 28042464 10.1039/c6sc01860b PMC5134683

[34] Pfeifer, V. et al. In situ observation of reactive oxygen species forming on oxygen-evolving iridium surfaces. *Chem. Sci.* **8**, 2143–2149 (2017). 28507666 10.1039/c6sc04622c PMC5407268

[35] Klingenhof, M. et al. High-performance anion-exchange membrane water electrolysers using NiX (X = Fe, Co, Mn) catalyst-coated membranes with redox-active Ni–O ligands. *Nat. Catal.* **7**, 1213–1222 (2024).

[36] Zhang, R. et al. First example of protonation of Ruddlesden–Popper Sr_2_IrO_4_: a route to enhanced water oxidation catalysts. *Chem. Mater.* **32**, 3499–3509 (2020).

[37] Wernet, P. et al. The structure of the first coordination shell in liquid water. *Science* **304**, 995–999 (2004). 15060287 10.1126/science.1096205

[38] Myneni, S. et al. Spectroscopic probing of local hydrogen-bonding structures in liquid water. *J. Phys. Condens. Matter* **14**, L213 (2002).

[39] Frati, F., Hunault, M. O. J. Y. & de Groot, F. M. F. Oxygen K-edge X-ray absorption spectra. *Chem. Rev.* **120**, 4056–4110 (2020). 32275144 10.1021/acs.chemrev.9b00439 PMC7227067

[40] Ping, Y., Nielsen, R. J. & Goddard, W. A. III The reaction mechanism with free energy barriers at constant potentials for the oxygen evolution reaction at the IrO_2_ (110) surface. *J. Am. Chem. Soc.* **139**, 149–155 (2017). 27936679 10.1021/jacs.6b07557

[41] Dickens, C. F., Kirk, C. & Nørskov, J. K. Insights into the electrochemical oxygen evolution reaction with ab initio calculations and microkinetic modeling: beyond the limiting potential volcano. *J. Phys. Chem. C* **123**, 18960–18977 (2019).

[42] Kuo, D. Y. et al. Influence of surface adsorption on the oxygen evolution reaction on IrO_2_(110). *J. Am. Chem. Soc.* **139**, 3473–3479 (2017). 28181433 10.1021/jacs.6b11932

[43] Tripathi, A., Ocampo-Restrepo, V. K., Norskov, J. K. & Kastlunger, G. Field effects explain the unintuitive potential response of electrochemical oxygen evolution in acid. *RSC Sustain.* **3**, 2659–2668 (2025).

[44] Kwon, S. et al. Facet-dependent oxygen evolution reaction activity of IrO_2_ from quantum mechanics and experiments. *J. Am. Chem. Soc.* **146**, 11719–11725 (2024). 38636103 10.1021/jacs.3c14271

[45] Willinger, E., Massué, C., Schlögl, R. & Willinger, M. G. Identifying key structural features of IrO_*x*_ water splitting catalysts. *J. Am. Chem. Soc.* **139**, 12093–12101 (2017). 28793758 10.1021/jacs.7b07079

[46] Petit, M. A. & Plichon, V. Anodic electrodeposition of iridium oxide films. *J. Electroanal. Chem.* **444**, 247–252 (1998).

[47] Mom, R. V. et al. Operando structure–activity–stability relationship of iridium oxides during the oxygen evolution reaction. *ACS Catal.* **12**, 5174–5184 (2022).

[48] Drevon, D. et al. Uncovering the role of oxygen in Ni-Fe(O_*x*_H_*y*_) electrocatalysts using in situ soft X-ray absorption spectroscopy during the oxygen evolution reaction. *Sci. Rep.* **9**, 1532 (2019). 30728373 10.1038/s41598-018-37307-x PMC6365557

[49] van der Heijden, O., Park, S., Eggebeen, J. J. J. & Koper, M. T. M. Non-kinetic effects convolute activity and Tafel analysis for the alkaline oxygen evolution reaction on NiFeOOH electrocatalysts. *Angew. Chem. Int. Ed.* **62**, e202216477 (2023). 10.1002/anie.202216477 PMC10108042 36533712

[50] Suntivich, J. et al. Estimating hybridization of transition metal and oxygen states in perovskites from O K-edge X-ray absorption spectroscopy. *J. Phys. Chem. C* **118**, 1856–1863 (2014).

[51] Grimaud, A., Hong, W. T., Shao-Horn, Y. & Tarascon, J. M. Anionic redox processes for electrochemical devices. *Nat. Mater.* **15**, 121–126 (2016). 26796721 10.1038/nmat4551

[52] Kasian, O., Grote, J.-P., Geiger, S., Cherevko, S. & Mayrhofer, K. J. J. The common intermediates of oxygen evolution and dissolution reactions during water electrolysis on iridium. *Angew. Chem. Int. Ed.* **57**, 2488–2491 (2018). 10.1002/anie.201709652 PMC5838529 29219237

[53] Scott, S. B. et al. The low overpotential regime of acidic water oxidation part I: the importance of O_2_ detection. *Energy Environ. Sci.* **15**, 1977–1987 (2022). 35706423 10.1039/d1ee03914h PMC9116083

[54] Righi, G. et al. On the origin of multihole oxygen evolution in haematite photoanodes. *Nat. Catal.* **5**, 888–899 (2022).

[55] Kresse, G. & Furthmüller, J. Efficient iterative schemes for ab initio total-energy calculations using a plane-wave basis set. *Phys. Rev. B* **54**, 11169–11186 (1996). 10.1103/physrevb.54.11169 9984901

[56] Kresse, G. & Hafner, J. Ab initio molecular dynamics for open-shell transition metals. *Phys. Rev. B* **48**, 13115–13118 (1993). 10.1103/physrevb.48.13115 10007687

[57] Kresse, G. & Joubert, D. From ultrasoft pseudopotentials to the projector augmented-wave method. *Phys. Rev. B* **59**, 1758–1775 (1999).

[58] Blöchl, P. E. Projector augmented-wave method. *Phys. Rev. B* **50**, 17953–17979 (1994). 10.1103/physrevb.50.17953 9976227

[59] Perdew, J. P., Burke, K. & Ernzerhof, M. Generalized gradient approximation made simple. *Phys. Rev. Lett.* **77**, 3865–3868 (1996). 10062328 10.1103/PhysRevLett.77.3865

[60] Dudarev, S. L., Botton, G. A., Savrasov, S. Y., Humphreys, C. J. & Sutton, A. P. Electron-energy-loss spectra and the structural stability of nickel oxide: an LSDA + *U* study. *Phys. Rev. B* **57**, 1505–1509 (1998).

[61] Ping, Y., Galli, G. & Goddard, W. A. III Electronic structure of IrO_2_: the role of the metal *d* orbitals. *J. Phys. Chem. C* **119**, 11570–11577 (2015).

[62] Hjorth Larsen, A. et al. The atomic simulation environment—a Python library for working with atoms. *J. Phys. Condens. Matter* **29**, 273002 (2017). 28323250 10.1088/1361-648X/aa680e

[63] Kumar, S. et al. An electrochemical flow cell for operando XPS and NEXAFS investigation of solid–liquid interfaces. *J. Phys.: Energy* **6**, 036001 (2024).

[64] Ravel, B. & Newville, M. ATHENA, ARTEMIS, HEPHAESTUS: data analysis for X-ray absorption spectroscopy using IFEFFIT. *J. Synchrotron Radiat.* **12**, 537–541 (2005). 15968136 10.1107/S0909049505012719

[65] Liang, C. et al. Data for article ‘Key role of oxidising species driving water oxidation revealed by time resolved optical and X-ray spectroscopies’. *Zenodo*10.5281/zenodo.18320343 (2026). 10.1038/s41563-026-02514-9 PMC13143823 41748930

