# Supplementary material for: Key role of oxidizing species driving water oxidation revealed by time-resolved optical and X-ray spectroscopies
Source: Nat Mater. 2026 Feb 26;25(5):799–807. doi: 10.1038/s41563-026-02514-9 (PMC13143823; doi:10.1038/s41563-026-02514-9)
Supplement: Supplementary file 1 — Supplementary Figs. 1–30, Notes 1–9 and Tables 1 and 2. [file 41563_2026_2514_MOESM1_ESM.pdf]

nature materials

Supplementary information https://doi.org/10.1038/s41563-026-02514-9

Key role of oxidizing species driving water oxidation revealed by time-resolved optical and X-ray spectroscopies

In the format provided by the authors and unedited

Contents

Supplementary Note 1 DFT Calculations 2

Supplementary Note 2 Structure of IrO_x 6

Supplementary Note 3 Optical absorption spectroscopy 8

Supplementary Note 4 Structural stability of IrO_x within spectroscopy measurement timescales 11

Supplementary Note 5 Operando near-edge X-ray absorption fine structure spectroscopy (NEXAFS) 16

Supplementary Note 6 Probing dynamic Ir oxidation/reduction at fixed energy 19

Supplementary Note 7 Electrochemical-mass spectrometry–Quantifying oxygen molecule release during OCP decay 26

Supplementary Note 8 Iridium dissolution during OCP decay measurements 32

Supplementary Note 9 Relation between oxygen evolution rate vs accumulated O^{-1} concentrations 34

Supplementary Note 1

DFT Calculations

Supplementary Table S1: Energy corrections used for each system. The values are given considering adsorbates on the 6 available cus and bri sites.

System	ZPE	∫CdT	TΔS	Total
$*H_2O_{cus}+*H_{bri}$	6.00	0.34	0.56	5.78
$*H_{bri}$	1.87	0.03	0.04	1.86
$*H_{cusi}$	1.40	0.03	0.04	1.39
$*H_{cus}+*H_{bri}$	3.27	0.06	0.07	3.25
$*OH_{cus}+*H_{bri}$	4.14	0.27	0.45	3.96
$*OH_{cus}$	2.23	0.27	0.46	2.04
$*O_{cus}$	0.44	0.19	0.33	0.30

As illustrated in Fig. S1 and Table S2, the inclusion of the Hubbard +U term does not alter the main insights from the calculations. Thus, for simplicity, the results in the main manuscript do not include this term. For the calculation of Bader charges[1], we increased the k-point mesh to 6x8x1, changed the convergence criteria for the electronic self-consistent iteration to 10^{-5} eV and doubled the number of grid points in the FFT grid along all lattice vectors. Fig. S1 also shows how the different surface states are chosen to represent the redox transitions. Table S2 shows that the change in average Bader charge related to redox transition 3 happens on the oxygen adsorbed on the Ir_{CUS} site, with the charges on O_{bri} atoms remaining almost unchanged.

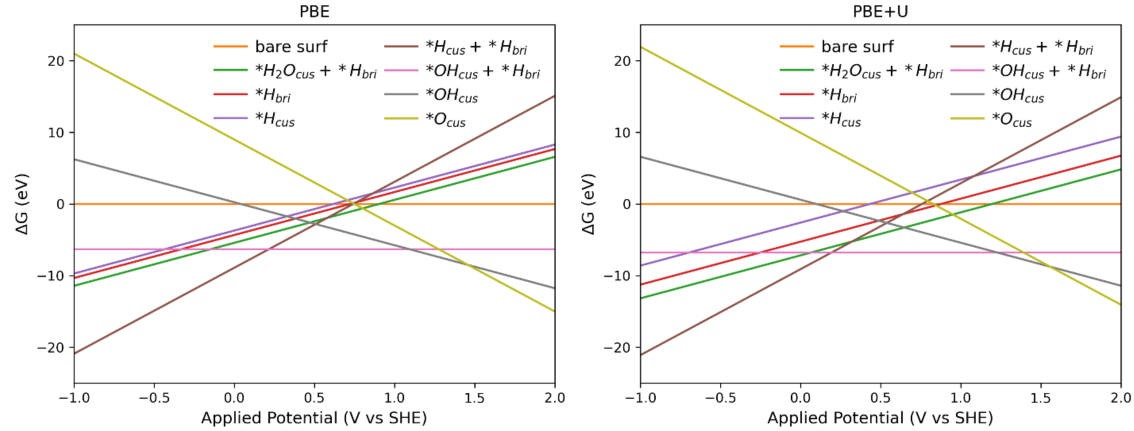

Figure S1 Surface Pourbaix diagrams of the IrO_2 surface calculated with PBE and PBE+U.

Supplementary Table S2: Average change in Bader charges for Ir_{cus}, Ir_{bri}, O_{cus}, and O_{bri} atoms present at the rutile IrO_2 surfaces during each redox reaction calculated with PBE and PBE+U, where positive (negative) numbers indicate an increase (decrease) in the number of electrons.

PBE				
	Ir_{cus}	Ir_{bri}	O_{cus}	O_{bri}
$*H_2O_{cus}+*H_{bri}$ -> $*OH_{cus}+*H_{bri}$	-0.43	0.13	0.15	0.06
$*OH_{cus}+*H_{bri}$ -> $*OH_{cus}$	-0.20	-0.07	-0.11	-0.06
$*OH_{cus}$ -> $*O_{cus}$	-0.04	0.05	-0.17	-0.04
PBE+U				
	Ir_{cus}	Ir_{bri}	O_{cus}	O_{bri}
$*H_2O_{cus}+*H_{bri}$ -> $*OH_{cus}+*H_{bri}$	-0.58	0.02	0.17	0.08
$*OH_{cus}+*H_{bri}$ -> $*OH_{cus}$	-0.09	-0.10	-0.07	-0.07
$*OH_{cus}$ -> $*O_{cus}$	0.01	0.04	-0.26	-0.05

Figure S2 shows the electronic density difference for the three redox transitions on rutile $IrO_2(110)$ surface. For all figures, the region in blue with no atom around represents the position of the missing H atom after the redox transition. As discussed in the Main Text, the Bader charge analysis shows that the first redox transition results in a large electron depletion near Ir atoms, while the O atom shows both electron depletion and accumulation regions. For the second redox transition, both Ir and O atoms show electron depletion and accumulation regions, which leads to the net depletion described through Bader charges. For the third redox transition, most of the electronic density decrease happens near the O atoms, while the Ir atoms show regions of electronic density depletion and accumulation.

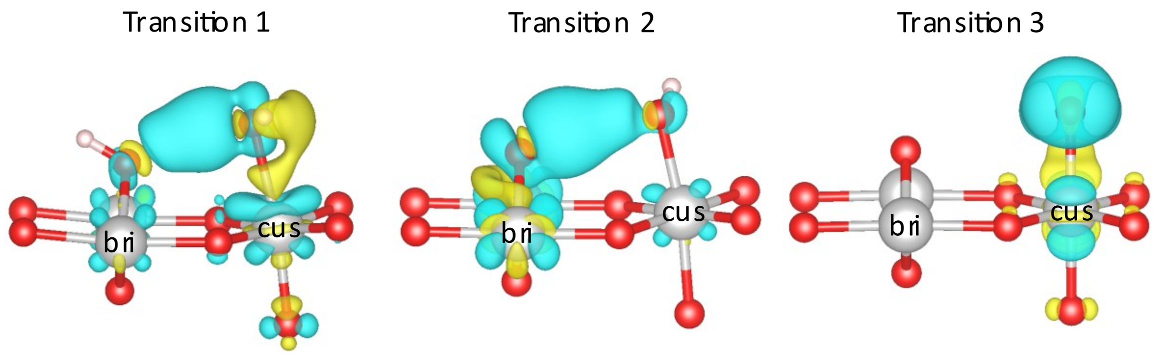

Figure S2: Electronic density difference for the three redox transitions at an isosurface value of 0.005 $e/Å^3$ where blue (yellow) represents a decrease (increase) of electron density due to the redox transition.

Redox transition 1: $* H_2O_{CUS} + *H_{Bri} \rightarrow *OH_{CUS} + *H_{Bri} + H^+ + e^-$, *Redox transition 2*: $*OH_{CUS} + *H_{Bri} \rightarrow *OH_{CUS} + *_{Bri} + H^+ + e^-$, *Redox transition 3*: $*OH_{CUS} \rightarrow *O_{CUS} + H^+ + e^-$

Figure S3 shows that despite the changes observed between rutile and hollandite surfaces, the main result from the Bader charges is still the same. When taken together, the first two redox transitions concentrate the electron depletion to iridium sites, while the third redox transition is the only one where electron depletion in oxygen sites is significantly larger.

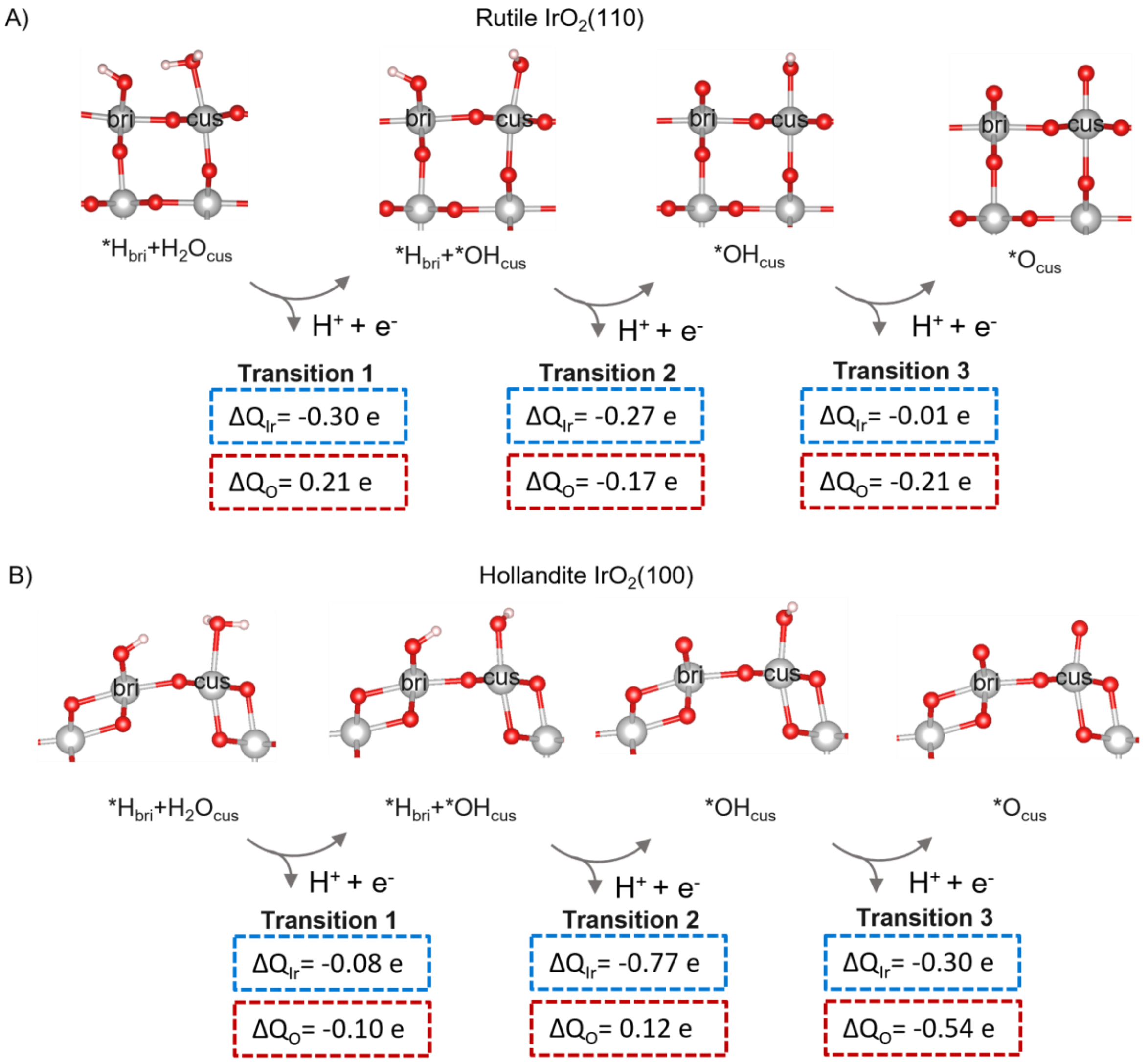

Figure S3 Structures involved in different redox transitions from DFT calculations for a) rutile (110) and b) hollandite (100) surfaces with the corresponding sum of average change in Bader charges for iridium and oxygen atoms present at the surfaces. The values represent the sum for coordinatively unsaturated sites (CUS) and sites with bridge oxygen bonded to two iridium atoms (bri), where positive (negative) numbers indicate an increase (decrease) in the number of electrons.

Fig. S4A-C shows the projected density of states for initial and final surface states of each redox transition. The states near the Fermi level are dominated by Ir states for the first redox transition. For the remaining redox transitions, both Ir (d) and O(p) states are present near the Fermi level, explaining why electron depletion from O atoms becomes more important at higher potentials. The band centers (Fig. S4D), of the occupied states were calculated as:

$$\varepsilon = \frac{\int E \, * \rho(E) \, dE}{\int \rho(E) \, dE}$$

where E is an energy value, ρ(E) is the density of state intensity at the value E, and the integral runs for all occupied states.

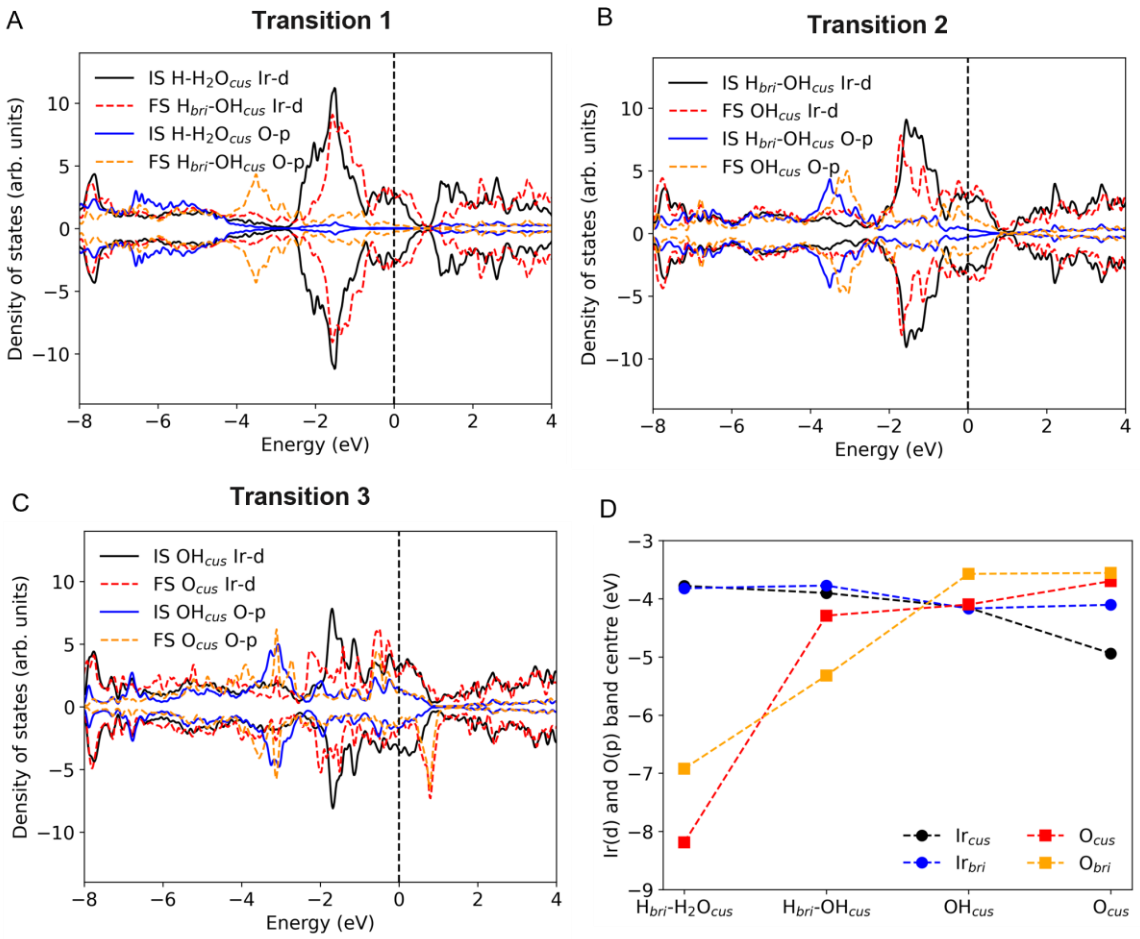

Figure S4: (A-C) Projected density of states (pDOS) for initial (IS) and final (FS) surface states of each redox transition. Black and red lines respectively show the Ir (5d) and O(2p) pDOS for the initial state, while blue and yellow lines show the Ir (5d) and O(2p) pDOS for the final state. (D) The d and p band centres for Ir and O are also presented separately for CUS and Bri sites.

Supplementary Note 2

Structure of IrO_x

TEM imaging was acquired using a Thermo Fisher Talos F200 electron microscope operating with a 200 kV acceleration voltage. To facilitate the transfer of the IrO_x film from the FTO to the holey carbon transmission electron microscopy (TEM) grid, the films were first wet with deionised water and then scraped to break the adhesion with the FTO. The TEM grids were then dragged across the FTO surface to transfer loose IrO_x film fragments to the holey carbon film. To minimise the cumulative electron fluence on the sample of interest, a sacrificial nanoparticle was used for initial lens alignment adjustments and focusing. An electron flux of 1 $e^- Å^{-2} s^{-1}$ was then administered to identify a region of interest, and the same electron flux was used to initially refine the focus. Additional focusing adjustments were made at higher magnifications with a flux of ca. 10 $e^- Å^{-2} s^{-1}$. A final electron flux of 100 $e^- Å^{-2} s^{-1}$ and a 1 s exposure time were used for acquisition, giving a total cumulative electron fluence of ca. 500 $e^- Å^{-2}$. The SAED patterns were acquired from an undamaged region of interest using the same instrumentation and a total electron fluence of < 100 $e^- A^{-2}$. Elemental mapping and quantification using energy dispersive spectroscopy (EDS) was acquired in scanning TEM (STEM) mode using the Super X detector system (0.9 srad) of the Thermo Fisher Talos F200 electron microscope.

Transmission electron microscopy (TEM) serves as a valuable tool for atomic-scale imaging of electrocatalysts. However, the susceptibility of iridium oxides to electron-beam damage necessitates careful monitoring of the cumulative electron fluence to ensure accurate structural characterisation. Limiting the total electron fluence to ca. 500 $e^- Å^{-2}$ supported the TEM acquisition in Fig. S5 without imposing any observable structural change. The lack of well-defined lattice fringes in the micrograph suggests no crystalline domain exists in the specimen and the absence of well-defined spots/rings of intensity in the fast-Fourier transform (FFT) or electron diffraction supports this conclusion.

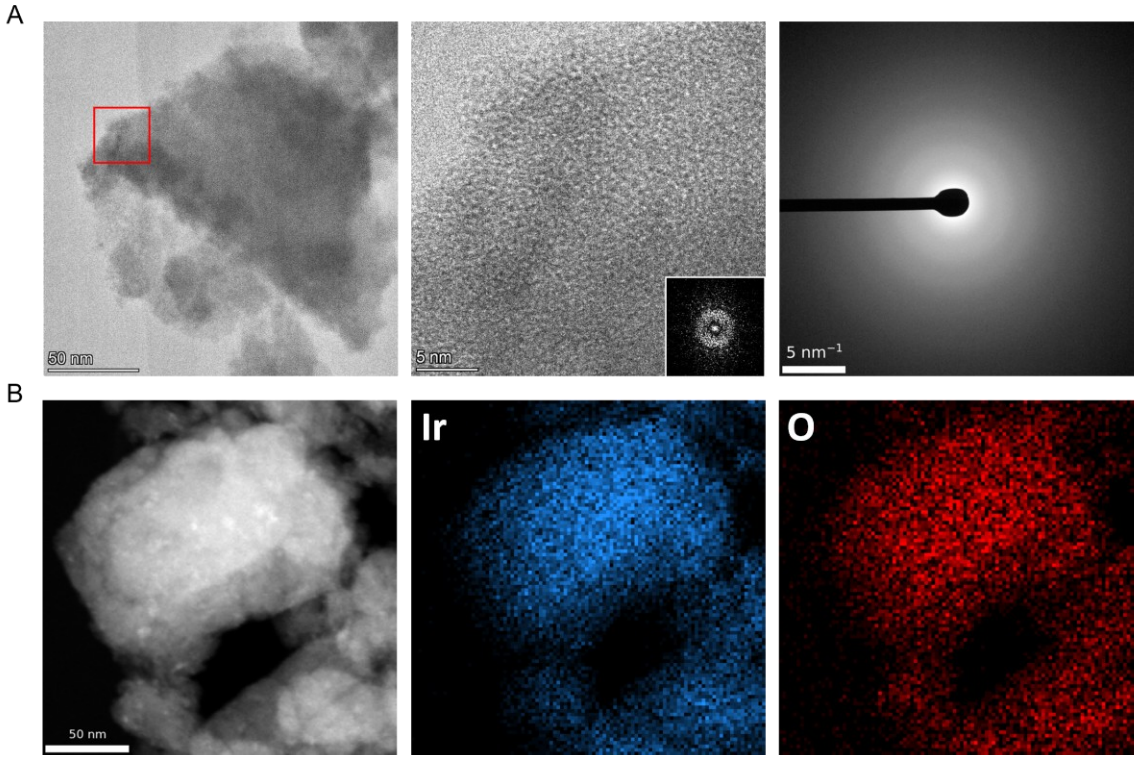

Figure S5 From left to right, the low-magnification TEM image, high-magnification TEM image and SAED. The red square in the left column TEM image denotes the region of interest shown at a higher magnification in the middle column. The inset in the middle column is the fast Fourier transform (FFT) of that image. No crystallinity was visible in any of the samples. (b) From left to right, the columns show a HAADF STEM image and the Ir and O STEM-EDS elemental maps.

Supplementary Note 3

Optical absorption spectroscopy

The optical spectroscopy used in this study is a difference spectroscopy, i.e. it measures the difference in optical absorption between two different redox states. For example, if we define different redox states as $State_A$, $State_B$, $State_C$ and $State_D$, the optical absorption of redox transitions is as follows:

- Redox transition 1 involves the transition of $State_A$ to $State_B$. The optical absorption measured experimentally is:

$$\Delta A_{redox\ transition\ 1} = \Delta A_{StateB} - \Delta A_{StateA}$$

- Redox transition 2 involves the transition of $State_B$ to $State_C$:

$$\Delta A_{redox\ transition\ 2} = \Delta A_{StateC} - \Delta A_{StateB}$$

- Redox transition 3 involves the transition of $State_C$ to $State_D$:

$$\Delta A_{redox\ transition\ 3} = \Delta A_{StateD} - \Delta A_{StateC}$$

Each redox transition therefore represents a *differential spectrum* between two adjacent redox states, rather than an absolute absorption. While the transitions exhibit broad absorption bands, each has a distinct spectral shape that can be extracted by subtracting adjacent spectra and normalising the resulting changes.

The methodology and associated programming scripts for spectral deconvolution were first established in our previous work.[2, 3] Briefly, the deconvolution process involves three key steps: (1) extraction of individual absorption spectra for distinct species via differential analysis; (2) application of linear combination fitting across spectra collected at all measured potentials to quantify the contribution of each species; and (3) experimental determination of extinction coefficients, followed by conversion of absorbance to species concentrations using the Lambert–Beer law. Detailed protocols for each step are provided in our earlier publications, and the open-source deconvolution scripts used for data analysis in this study are available (https://github.com/Caiwu-L/Paper_amorphous-IrOx_vs_Rutile_IrO2). The resulting spectra and deconvolution outcomes from this study are presented below:

The resulting deconvoluted characteristic spectra for different redox transitions are shown in Fig S7, also consistent with our previous report.[2, 3] The linear combination fitting results are shown in Figure S8. Thus, the optical nature of redox transition 3 is the characteristic differential spectrum associated with the $State_C \rightarrow State_D$ transition, as shown in red in Fig S7. This feature becomes evident only at potentials above ~1.3 V_{RHE} and has a dominant feature near 500 nm. The physicochemical nature of

each redox transition is interpreted by comparison of optical spectroscopies and Ir-L edge and O-K edge absorption spectroscopies.

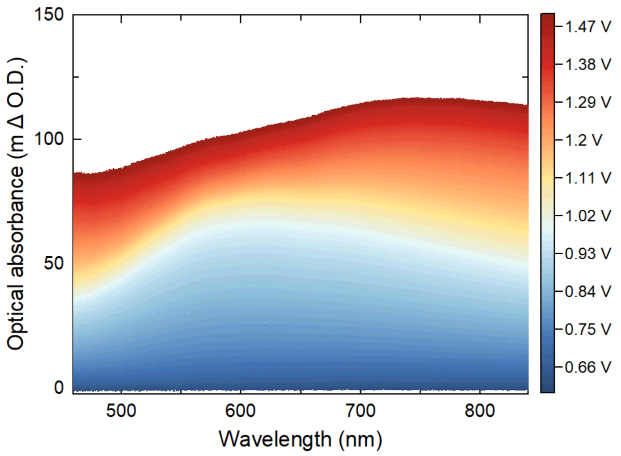

Figure S6 Absorption spectra of amorphous IrO_x in 0.1 M $HClO_4$ during a linear sweep scan from 0.6 V to 1.5 V_{RHE} and 0.5 to 1.5 V_{RHE}, respectively, at a scan rate of 1 mV s^{-1} (iR corrected). Absorption changes were recorded at every 1 mV. The absorption changes are calculated with respect to the absorption at 0.6 V.

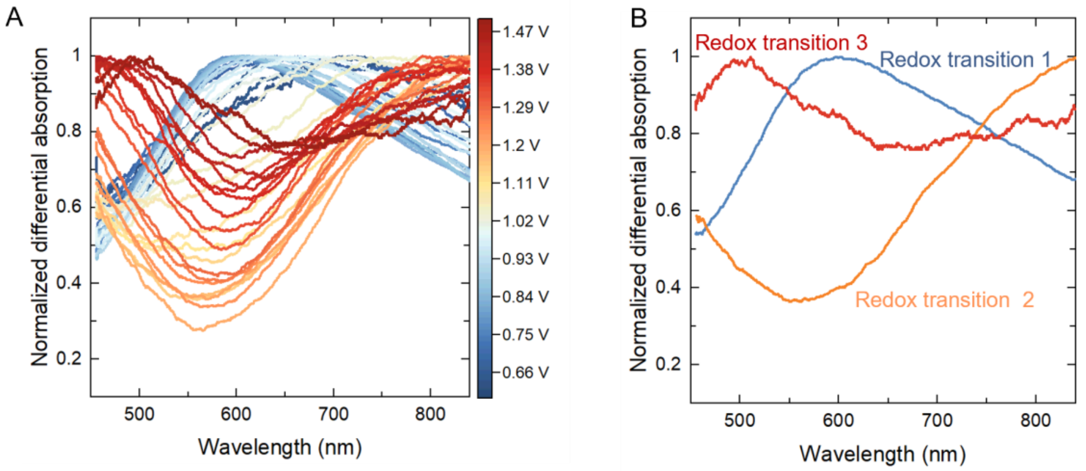

Figure S7 A) Differential analysis of absorption spectra for IrO_x at every 20 mV. The differential absorption spectra are obtained by subtracting adjacent spectra and normalizing the maximum absorbance to unity, representing a change in absorbance that corresponds to a potential change of 20 mV. B) Extracted spectra for individual redox transitions in amorphous IrO_x. The absorption spectra for redox transition 1 (light blue), 2 (light yellow) and 3 (red) in amorphous IrO_x are extracted from the potential regime where the shapes of the spectra in A are invariant, representing consistent individual redox processes. These correspond to differential spectra between 0.8 and 0.78 V_{RHE}, 1.18 and 1.16 V_{RHE}, and 1.5 and 1.48 V_{RHE}, respectively.

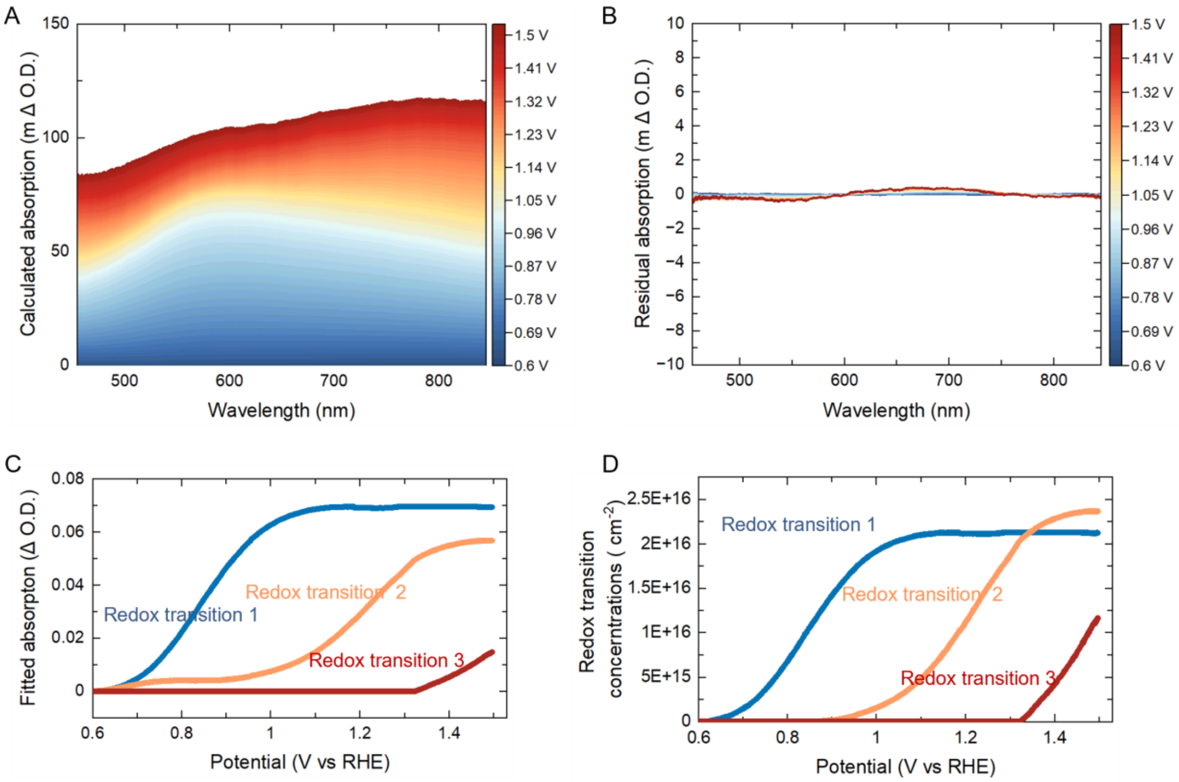

Figure S8. Deconvolution result for IrO_x in 0.1 M $HClO_4$. (A) Calculated differential absorption spectra of IrO_x in acid at every 1 mV during a linear sweep scan from 0.6 V to 1.50 V_{RHE}. (B) Fitting residuals between calculated spectra and experimental spectra at each potential. (C) Calculated absorption at the peak wavelength of each redox transition as a function of potential. (D) Densities of redox transitions for IrO_x that have occurred as a function of the potential. The conversion of absorption into the densities of redox transitions is based on the measured extinction coefficient obtained from our previous works[2, 3]

Supplementary Note 4

Structural stability of IrO_x within spectroscopy measurement timescales

Previous work has shown that amorphous IrO_x can transform to rutile under PEMWE conditions,[4] which could also contribute to the spectroscopic changes observed in our measurements. To assess the structural stability of the IrOx film and the impact of electrochemical testing on structure under our spectroscopic conditions, we performed 20 cyclic voltammetry (CV) cycles between 0.6 and 1.5 V_{RHE} on IrO_x, and a series of chronoamperometric holds at 0.25, 0.5, 1.0, 1.5, 2.0, 2.5, and 3.0 mA cm^{-2}_{geo} for 10 minutes each (total duration: 2 hours 10 minutes), following a protocol similar to that used in our X-ray absorption spectroscopy measurements. The structural evolution of the film after these electrochemical protocols was examined by TEM and Selected Area Electron Diffraction (SAED) and energy dispersive X-ray spectroscopy (EDS).

Fig. S9 shows that the electrochemical response remains consistent over 20 CV cycles, with only minor differences observed in the first cycle, likely due to surface cleaning and improved wetting. In addition, the OER potential remains stable during 2 hours of chronoamperometric holding at current densities ranging from 0.25 to 3 mA cm^{-2}_{geo}. The structure changes of IrO_x after these tests were examined using TEM and SAED, using the same method described in Supplementary Note 2, with measurements on a pristine sample (adapted from Figure S5) from the same deposition batch used as a reference.

TEM imaging and electron diffraction acquired for the pristine, cycled and constant-current samples are shown in Fig. S10a, 10b and 10c, respectively. In all cases, TEM imaging reveals no well-defined lattice fringes, and the absence of well-defined spots/rings of intensity in the fast Fourier transform (FFT) suggests no crystallinity is present. This is further supported by the SAED patterns acquired for the same samples, which showed no well-defined diffraction rings or spots in any of the films, as would be expected for a crystalline structure. The structural characterisations presented here underline that no change in structure is seen for the electrodeposited IrO_x after the applied electrochemical procedures.

Elemental mapping of the three films was undertaken in order to probe the distribution of Ir and O species in the pristine and electrochemically tested samples. High-angle annular dark-field (HAADF) STEM images of three regions of interest and the corresponding STEM EDS Ir and O STEM EDS elemental maps are presented in Figures S11a, 11b and 11c for the pristine, cycled and constant-potential samples, respectively. For the pristine sample and tested films, uniform mixing of Ir and O was present with no visible inhomogeneity in Ir and O, suggesting the stability of the oxide structure with respect to the electrochemical procedures.

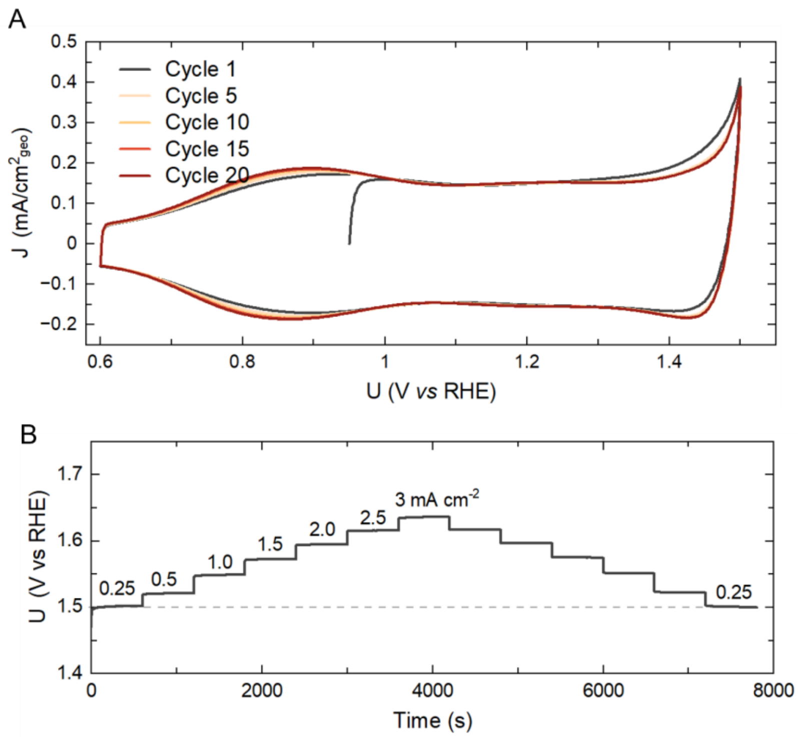

Figure S9 A) CVs of IrOx for cycle 1, 5,10, 15 and 20 at a scan rate of 10 mV s^{-1} in 0.1 M $HClO_4$ electrolyte. (B) Chronopotentiometry of IrO_x from 0.25 to 3 mA cm^{-2}_{geo}.

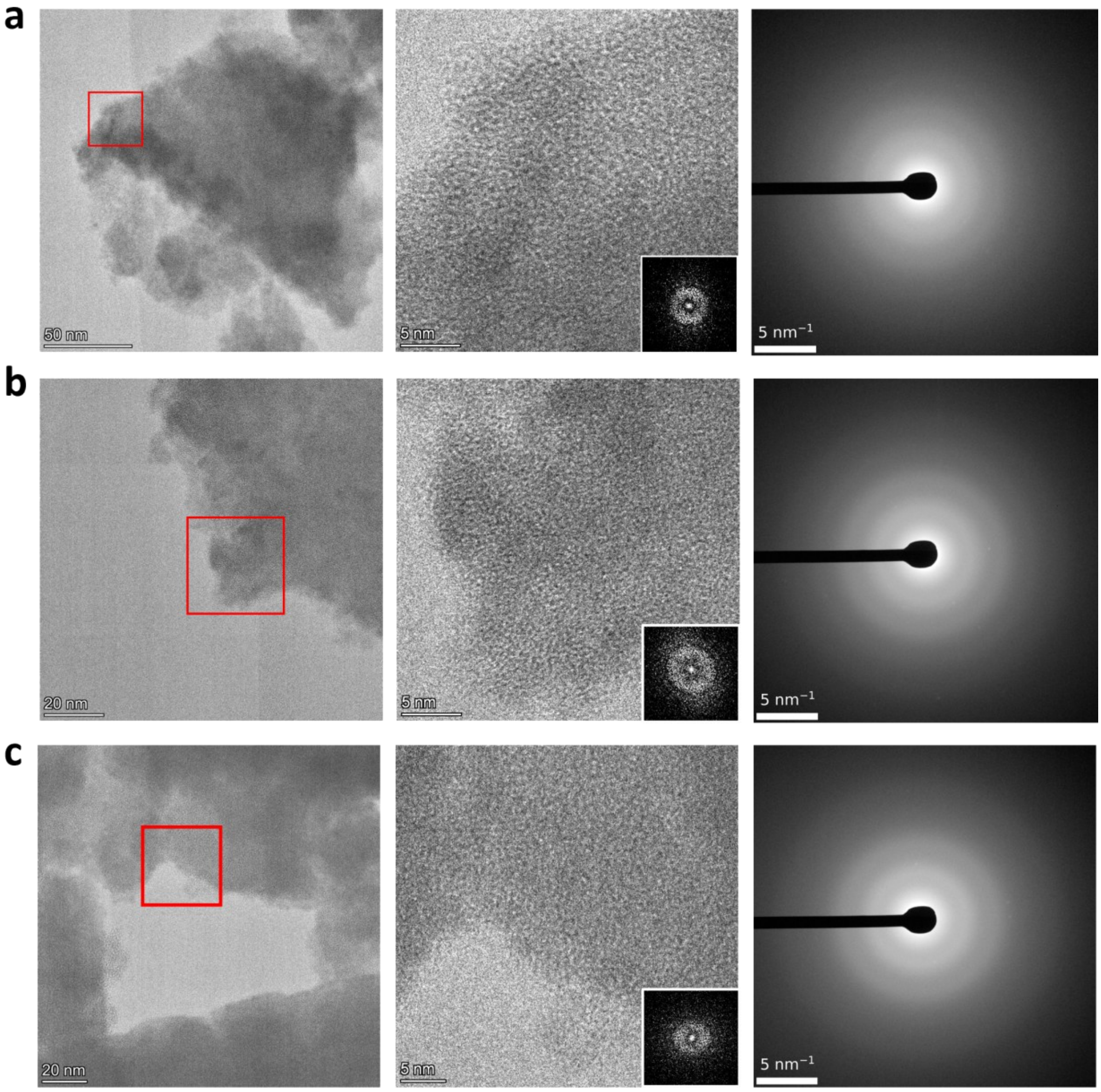

Figure S10 From left to right, the columns show: low magnification TEM; high magnification TEM; and SAED; plotted for (A) the pristine, (B) the cycled and (C) the constant potential samples. The red squares in the left column TEM image denote the region of interest shown at a higher magnification in the middle column. The insets in the middle column are the fast Fourier transform (FFT) of that image. No crystallinity was visible in any of the samples.

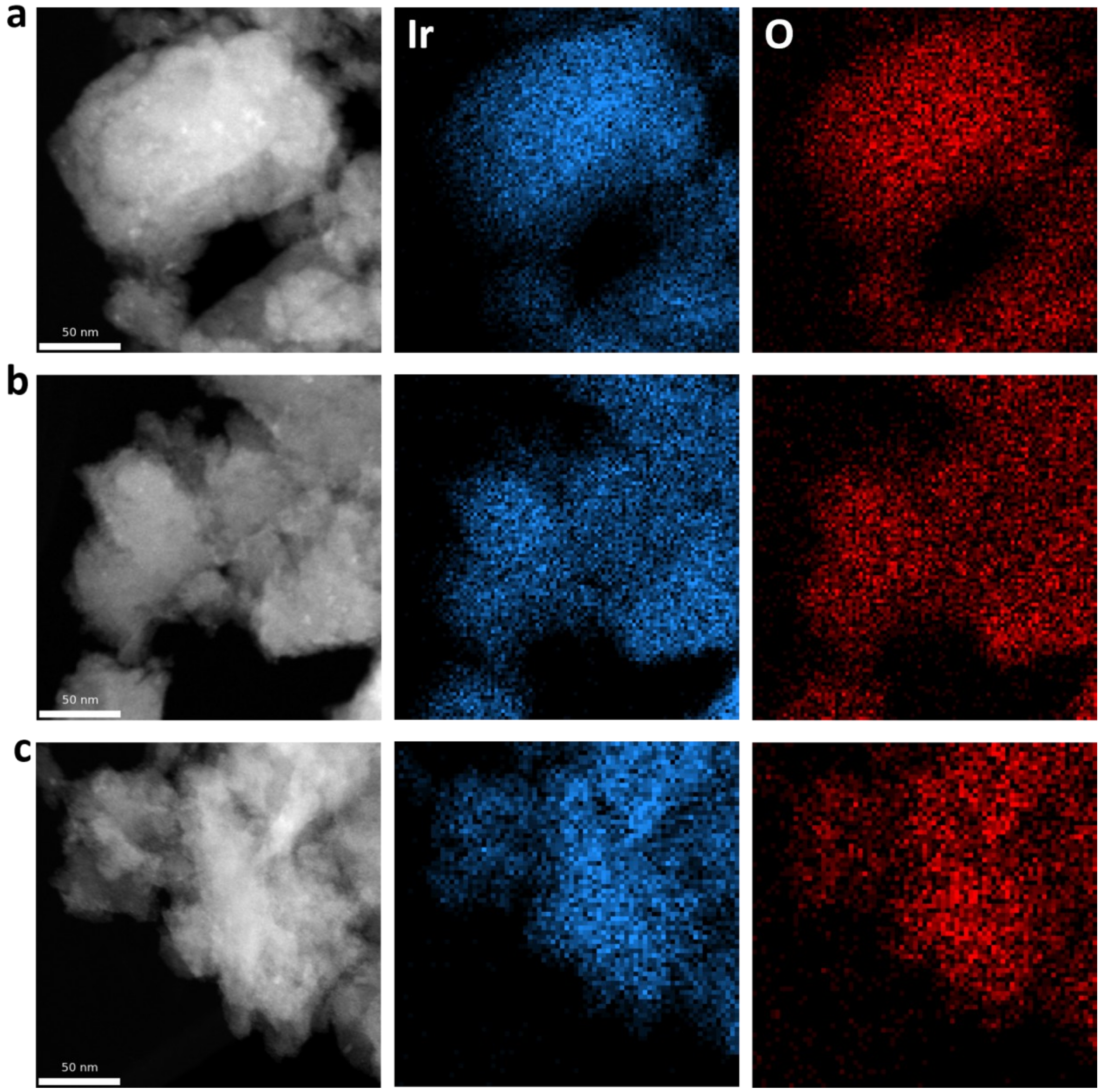

Figure S11 From left to right, the columns show a HAADF STEM image and the Ir and O STEM-EDS elemental maps for (A) the pristine, (B) the cycled and (C) the constant potential samples.

The surface state changes under similar conditions, as well as the reversibility and stability of the film during cycling, were previously analysed by XPS (Fig S12) and operando optical spectroscopy (Fig. S13) in our work,[2] and the results are adapted here for comparison. The XPS results show that the Ir 4*f* and O 1s spectra taken before and after CV cycles overlap, suggesting similar surface states before and after the measurement. The optical absorption spectra during a reverse potential scan from 1.48 V_{RHE} to 0.66 V_{RHE} for IrO_x are almost identical to those observed in a forward scan from 0.66 to 1.48 V_{RHE}, suggesting the absorption behaviour is highly reversible with potential. Moreover, the optical spectroscopy measurements after 20 cycles show that the spectra obtained are reproducible over 20 CVs. Together with the electrochemical results and structural analyses, these findings indicate that no significant structural changes occur in IrO_x within the timescales and conditions of our measurements.s

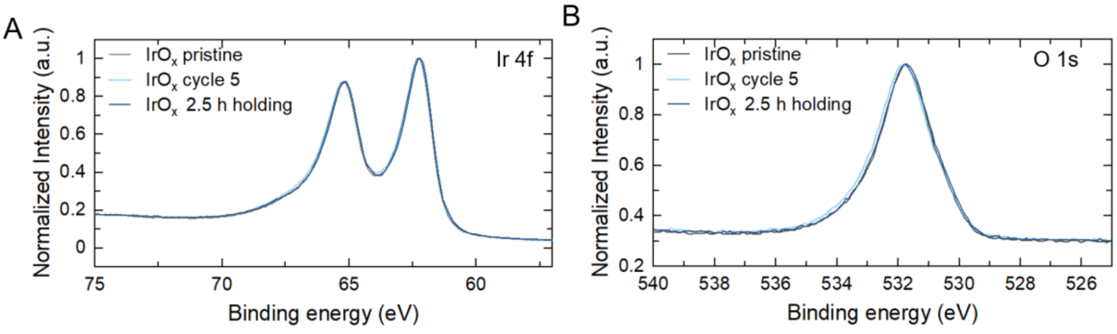

.

Figure S12 Comparison of XPS spectra before and after CV cycles and constant current holding electrochemical measurement. (A) Ir 4f spectra and (B) O 1s spectra of IrO_x, The data were reproduced from our previous work.[2]

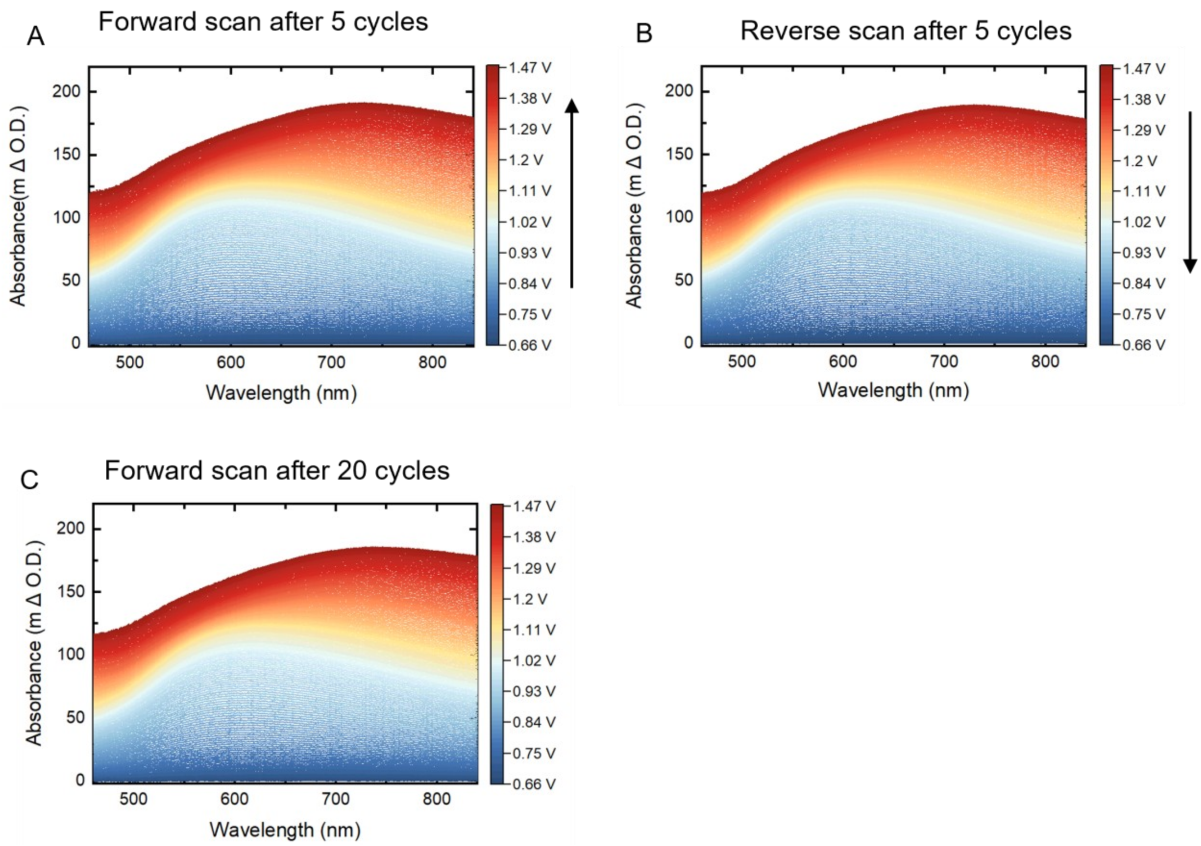

Figure S13 (A) Differential absorption spectra of amorphous IrO_x during a linear sweep scan from 0.66 V_{RHE} to 1.48 V_{RHE} in 0.1M $HClO_4$ at a scan rate of 5 mV s^{-1}. Absorption at 0.66 V_{RHE} is taken as reference absorption (B) Differential absorption spectra during a reverse scan from 1.48 V_{RHE} to 0.66 V_{RHE} of the same IrO_x sample. Absorption at 0.66 V_{RHE} is taken as the reference absorption. c) Differential absorption spectra of amorphous IrO_x after 20 cycles of CV. The data was reproduced from our previous work.[2]

Supplementary Note 5

Operando near-edge X-ray absorption fine structure spectroscopy (NEXAFS)

Fig. S15 compares the normalised O-K-edge spectra for amorphous IrO_x deposited on a gold-coated SiN_x substrate with a standard rutile IrO_2 powder (Alfa) attached to carbon tape. The O-K-edge spectra for the substrate were also measured as a control. SiN_x substrate shows negligible absorption in the O-K-edge region (Fig. S16), while carbon tape has oxygen absorption features only at an energy higher than 531 eV. In the lower energy region between 527 to 531 eV, both amorphous IrO_x and the standard IrO_2 show clear absorption peaks, which are assigned to electronic transitions from O 1s into the hybridization of O 2p and Ir 5d t_{2g} states in previous study.[5] Amorphous IrO_x exhibits a broader and less intense peak in the 528 to 530.5 eV range, in contrast to rutile IrO_2, which features a sharp peak at approximately 530.2 eV. This discrepancy aligns with the findings of Pfeifer et al.,[6, 7] suggesting that the additional absorption below 530 eV in amorphous IrO_x can be attributed to oxygen that has less bonding than 3 Ir, compared to the dominant bulk O^{2-} in rutile IrO_2, which absorbs around 530 eV. The barely observed peaks at around 530 eV indicate there is barely μ-3 lattice oxygen, confirming it's very short and highly amorphous nature. The measurements in total electron yield (TEY) mode, which offers enhanced surface sensitivity[8], also produced spectra that closely resemble those from total fluorescence yield (TFY) mode, further confirming the uniform oxygen species in IrO_x .

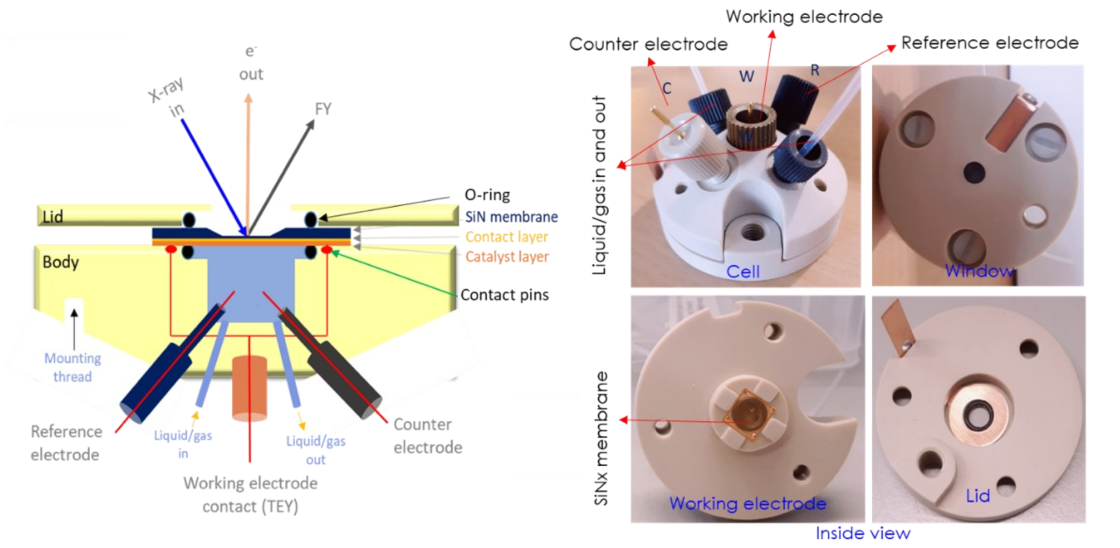

Figure S14 Left: schematic showing the structure of the cell designed. Right: the image of the cell used. WE CE and RE represent the working electrode, counter electrode and reference electrode. These cell design was adapted from previous work by Kumar et al.[9].

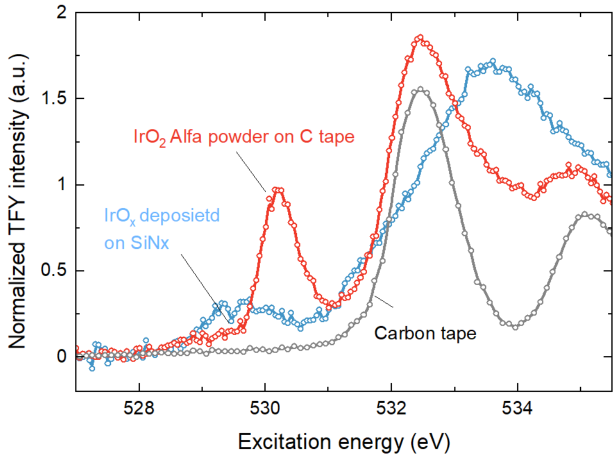

Figure S15 Comparison of the O K-edges of amorphous IrO_x deposited on gold-coated SiN_x substrate and commercial rutile-type IrO_2 powder (Alfa). The amorphous IrO_x shows an additional pre-edge feature at around 529.2 eV and less intensity at 530.2 eV, where rutile IrO_2 has a sharp resonance. The data is normalized to the fluorescence at the post edge at ~550 eV

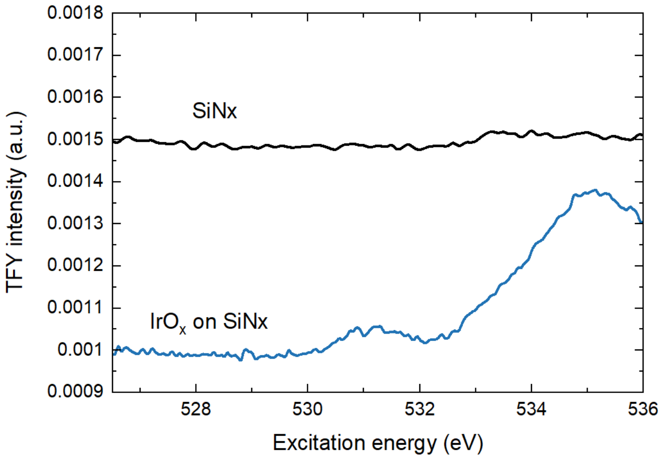

Figure S16 Comparison of the O K-edges date of amorphous IrO_x deposited on gold coated SiN_x substrate and the bare substrate as a control.

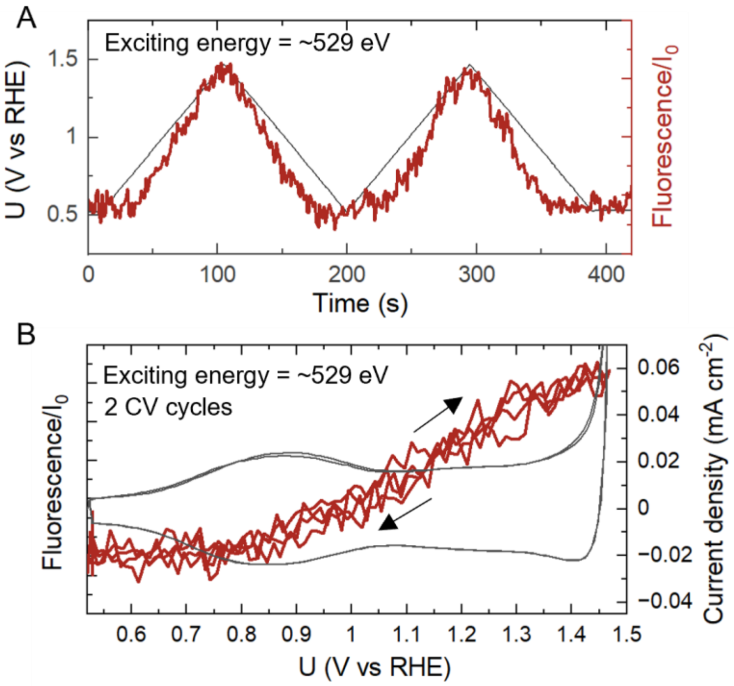

Figure S17 (a) The O-K-edge NEXAFS fluorescence intensity changes at a fixed exciting energy of ~529.0 eV across two cycles of CVs from 0.5 V_{RHE} to approximately 1.47 V_{RHE} at a scan rate of 10 mV s^{-1}, in 0.1 M $HClO_4$ electrolyte. (b) Comparing the current density and the change in fluorescence intensity at ~529.0 eV as a function of potential, as derived from (a).

Supplementary Note 6

Probing dynamic Ir oxidation/reduction at fixed energy

In time-resolved X-ray spectroscopy, the fluorescence intensity is continuously monitored at a fixed beam energy while performing electrochemical measurements. Fig. S18a shows the white line peak position of XANES spectra shift to higher energy with increasing potential, suggesting higher oxidation states. This shift in peak position leads to a decrease in intensity at energy before peak (as shown by dash line at 11218 eV), an increase at energy after peak (as shown by the dashed line at 11222 eV) and a negligible change at energy of post edge (energy that beyond Ir-L_3 edge). Fig. S18b illustrates how the fluorescence intensity changes at specific energies (11218, 11222, and 12150 eV) across two cycles of CV from 0.5 V_{RHE} to approximately 1.44 V_{RHE}. The intensity variations at fixed energies of 11218 and 11222 eV closely follow the applied potential, while the intensity at 12150 eV is independent of potential, corroborating the observations from Fig. S18a. Fig. S18c further shows the fluorescence intensity at 11222 eV changes as a function of potential for two 2 CV cycles. It is clear that the oxidation and reduction of iridium are highly reversible, with the forward scan almost identical to the backward scan within 2 cycles. Furthermore, the increase in fluorescence signal with rising potential becomes less pronounced at high potential (>1.4V_{RHE}). These changes in fluorescence intensity can be converted to oxidation state using a calibrated relation between the raw intensity at a given energy and the oxidation states, which is determined from the peak position of XANES spectra.

Fig. S19a compares the Ir average oxidation state and fluorescence at 11222 eV, as a function of potential. The trends of increase in fluorescence closely align with that of Ir oxidation states. This leads to an almost linear correlation of oxidation state and fluorescence intensity at 11222 eV (Fig. S19b), with a fitted relation as follows:

$$\text{Fluorescence} = 0.004 \times \text{Ir oxidation state} - 0.0058 \tag{2}$$

This equation suggests that for each unit increase in oxidation state, the fluorescence intensity at 11222 eV increases by around 0.004. However, it is worth noting that this linear relationship, while generally holds true across experiments, the exact parameters in the fitted linear equation are specific to the measurements conducted at the same spot of the sample, same beamline and at the same period of beamtime. This is because the raw fluorescence intensity at fixed energy could be affected by factors that are unrelated to oxidation states, including variations in samples, probe positions and beam energy source. Therefore, while we discuss the fluorescence data in relation to oxidation states, one should caution against directly converting fluorescence intensity to specific oxidation states without considering these variables. A similar method combining electrochemistry with time-resolved XAS was employed by Dau and co-works[10] to track oxidation-state changes in cobalt catalysts.

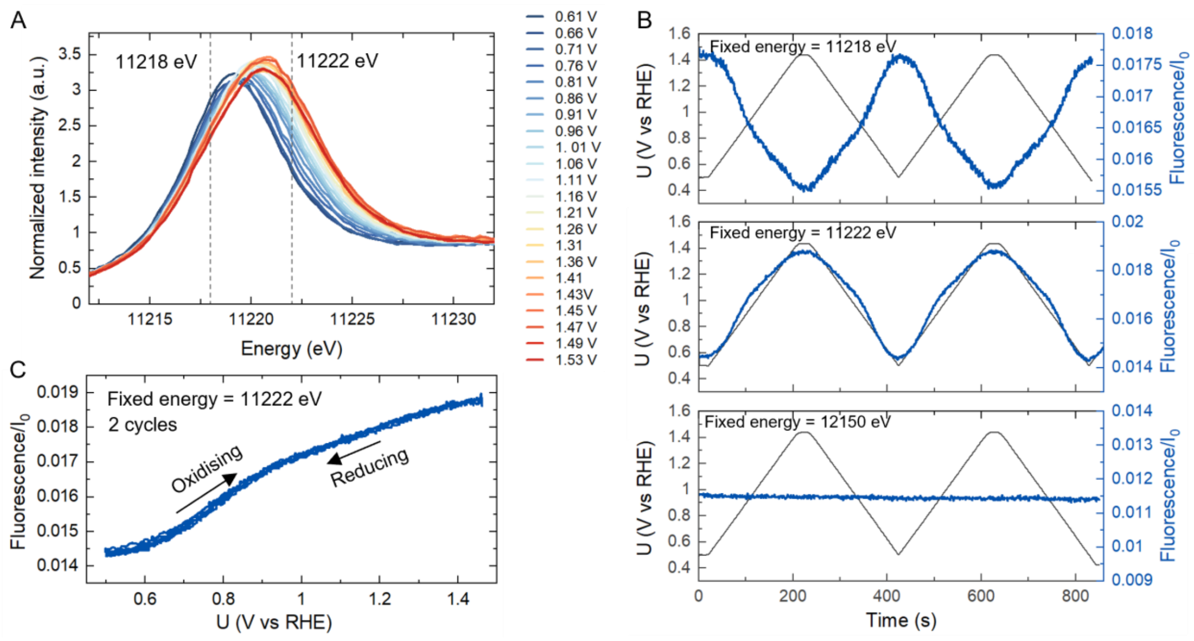

Figure S18 (A) Ir L_3-edge XANES region of IrO_x on FTO measured at different potentials in 0.1 M $HClO_4$, adapted from Fig. 2B in the main manuscript. Dashed lines indicate the intensity change at a fixed energy of 11218 eV and 11222 eV. (B) The fluorescence intensity changes at specific energies (top- 11218 eV, middle-11222 eV, and bottom-12150 eV) across two cycles of cyclic voltammetry (CV) from 0.5 V_{RHE} to approximately 1.44 V_{RHE} at a scan rate of 10 mV s^{-1}, in 0.1 M $HClO_4$ electrolyte. (C) change of fluorescence intensity at 11222 eV as a function of potential, obtained from the middle panel of (B)

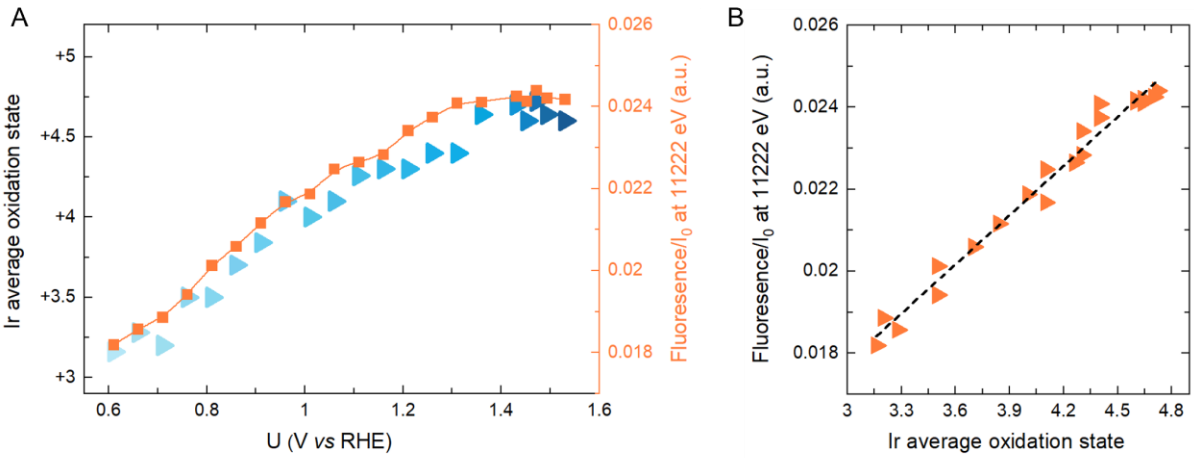

Figure S19 (A) Comparison of average iridium oxidation state (left y-axis) with fluorescence at a fixed energy of 11222 eV (right y-axis) as a function of potential. (B) Correlation between fluorescence at 11222 eV and average Ir oxidation state. The dashed line indicates a linear regression fit of the data.

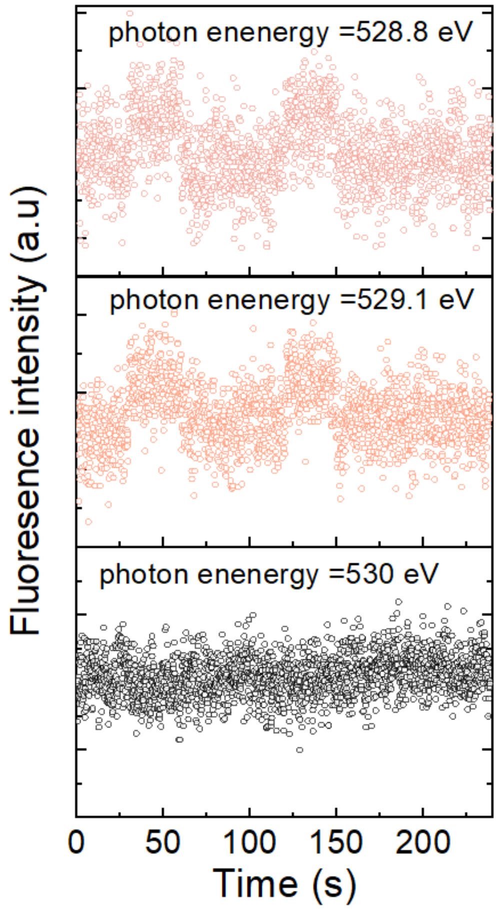

Figure S 20 Change in oxygen fluorescence intensity at different exciting energy for step potential measurements from 1.1 to 1.27 V_{RHE}.

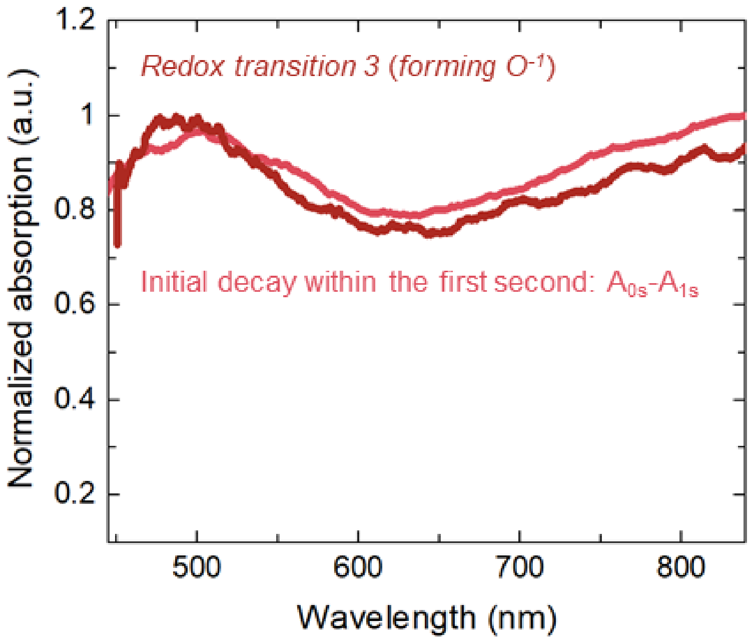

Figure S21 Comparison of differential spectra obtained during OCP decay and redox transitions from linear sweep voltammetry. Differential absorption spectra collected within the first 1 s of OCP decay (light red) are compared with the differential absorption features of redox transition 3 obtained from spectral deconvolution (dark red). All spectra are normalised to their maximum absorbance for comparison. Data are adapted and replot from our previous work.[2]

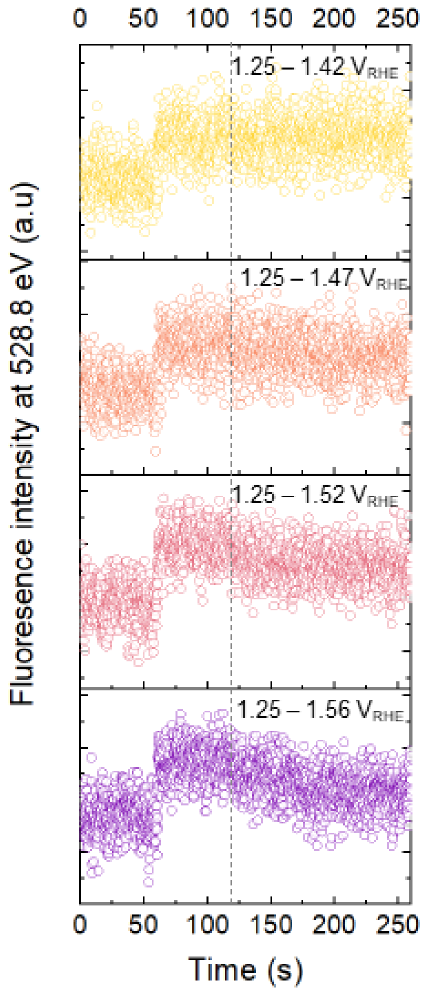

Figure S22 Change in oxygen fluorescence intensity at 528.8 eV during open circuit potential decay measurement at different applied potential. The decay rate of signal increase with increasing potential.

Supplementary Note 7

Electrochemical-mass spectrometry—Quantifying oxygen molecules release during OCP decay

The oxygen signal is measured in two sequential procedures. The first measurement- open circuit potential (OCP) decay, involved stepping up the potential from 1.4 V_{RHE} to 1.46 V_{RHE}, holding it for 10 s, then switching to open circuit to allow the redox states accumulated at high potential to relax naturally. The second measurement, step potential measurement, served as a control measurement for OCP decay. After holding potential at 1.46V_{RHE} for 10 s, the potential is stepped back to 1.4 V_{RHE} to actively reduce the redox states accumulated at 1.46 V_{RHE} to the same level of 1.4 V_{RHE}. These measurements were performed in sequence on the same sample without interruption, differing only in how the potential was returned to its starting level – either through an external circuit or by switching to an open circuit to allow natural relaxation. As shown in Fig. S23a, distinct increase in O_2 signal is observed when the potential raised from 1.4 V_{RHE} to 1.44 V_{RHE}, which then gradually returned to its baseline level after stepping back the potential to 1.4 V_{RHE}. The delay in the return of oxygen signal back to baseline is attributed to the diffusion time required for oxygen to move from the electrode surface to the mass spectrometer, varying from a few seconds to tens of seconds and depend on the oxygen concentration.[11, 12] Interestingly, following this step potential measurement, an open circuit decay measurement shows a significantly larger oxygen signal in comparison. This indicates the release of additional oxygen during the open circuit decay compared to step potential measurements. To eliminate the possibility of a time-dependent artefact or irreversible change in materials on oxygen signal recording, the sequence of experiments was reversed, performing an open circuit decay measurement before the step potential experiment. The results, as shown in Fig. S23b, consistently exhibit increased oxygen release during open circuit decay, confirming the observation that extra oxygen is released during open circuit potential.

To quantify the additional oxygen released during open circuit decay, we calibrated EC-MS signals to the actual amounts of oxygen using the relation of OER current and oxygen signal on a clean polycrystalline Pt surface. Based on previous findings from Cherevko, Mayrhofer and co-workers[13], Pt shows minimal dissolution under constant current operation after ~1000 s. Thus, by pre-stabilizing Pt surface to minimize the dissolution, we can assume an OER Faraday efficiency of approximately 100% on Pt. This assumption allows us to use the charge passed through the reaction to determine the amount of O_2 produced, thereby correlating it with the ECMS-detected signal. Fig. S24a illustrates the result of calibration experiment, where the current over specific periods (shown as dark rectangular area) are integrated to charge and compare with the EC-MS detected signal. Fig. S24b illustrates a linear correlation between the electrochemical mass spectrometry (EC-MS) detected signal, measured in nanocoulombs (nC), and the quantity of oxygen produced, quantified in nanomoles (nmol). This linear relationship yielded a calibrated sensitivity factor, represented by the fitted slope, of approximately

0.109 nC/nmol. This sensitivity factor enabled us to accurately convert EC-MS signals into oxygen amounts for all subsequent experiments, providing a clear and direct method for quantifying oxygen release.

The amount of extra oxygen release during open circuit decay is calculated by comparing the detected oxygen between step potential and open circuit potential decay measurement. To enhance the visibility of the oxygen signal decay during open circuit, we extended the potential holding time for both measurements from 10 seconds to 60 seconds before shifting to open circuit (Fig. S24c). This led to a more stable oxygen flux in the mass spectrometer, providing a clearer baseline from which the subsequent decay could be more distinctly observed. The potential, current and oxygen signal data for the two measurements are then overlapped for comparison (Fig. S24d). Prior to 120 seconds, the potential and current were almost identical in both operations. However, differences emerged after 120 seconds. During step potential, a cathodic current peak corresponding to the reduction of accumulated states was observed, while in open circuit potential decay, no current was recorded. Between 120 and 330 seconds, there was a noticeable increase in oxygen release during the open circuit decay measurements compared to that of step potentials measurements. This increase could not be solely attributed to the diffusion of oxygen produced between 60 and 120 seconds. The disparity in oxygen levels over time is further illustrated in the bottom panel of Fig. S24d, showing a net oxygen production of approximately 0.698 nmol cm^{-2} during open circuit decay (as calculated by integrating the O_2 signal from 120 to 330 seconds).

The same phenomenon was observed for different applied potential steps including potential steps 1.4-1.42 V_{RHE}, 1.4-1.42 V_{RHE}, 1.4-1.45 V_{RHE}, 1.4-1.46 V_{RHE}, 1.4-1.47 V_{RHE} and 1.4-1.48 V_{RHE} (Fig. S25 and Fig. S26). As step potential increases, the net amount of oxygen produced during open circuit potential clearly increases.

To further understand this link, we quantify the concentration of O^{-1} at different potentials and compare it with the corresponding amount of oxygen produced during open circuit potential decay. The concentration of O^{-1} is inferred from the integration of cathodic current peak observed when the potential is stepped back, representing the charge required to revert all high-potential-accumulated O^{-1} back to its O^{-2} state at start potential. We note that the difference between open circuit decay and step potential measurements is negligible for potentials lower than approximately 1.415 V_{RHE}. Therefore, the potential range starting from 1.415 V_{RHE} was selected for charge integration. This ensures that we are examining the charge specifically related to active O^{-1} that can produce oxygens molecule while minimizing influences from charges associated with high oxidation iridium sites inactive towards the OER or those species contributing to oxygen release on a timescale longer than the experiment period or below the detection limit of EC-MS. The integration of charge at different potentials is shown in Fig. S27. These integrated charge densities are used as O^{-1} concentration and compared to the oxygen

amount detected in open circuit decay. We also note that, the integrated cathodic charge in our pulsed-potential measurements is assumed to arise predominantly from the discharge of O^{-1} species, while the contribution from double-layer charging is neglected. This assumption is supported by both our experimental observations and previous literature. We performed Cyclic voltammetry (CV) measurement at potentials well below the potential window of redox processes, where only double-layer charging is expected. As shown in Fig. S28, the current density decreases sharply below 0.4 V. A simple estimation shows that the current density at 0.3 V (0.0024 mA cm^{-2}) is more than an order of magnitude smaller than that at 1.2 V (0.069 mA cm^{-2}). Assuming the current at 0.3 V arises solely from double-layer charging, its contribution would be less than 5% of the total charge. This finding is consistent with the recent study by Travis Jones *et al.*,[14] who compared the capacitance of five different IrO_x samples using four independent methods: (1) CV charge integration, (2) current-decay fitting (3) scan-rate dependence analysis, and (4) impedance spectroscopy, and found that the pseudocapacitance of IrO_x is one to two orders of magnitude higher than the double-layer capacitance predicted from the BET surface area of nanoparticles, suggesting that charge on iridium oxides is dominated by redox process. Based on these analyses, we take the view that the majority (> ~95%) of the cathodic charge measured in our pulsed-potential experiments originates from the reduction of O^{-1}, with only a minor contribution from double-layer discharge. For simplicity, we therefore use the integrated cathodic charge as a measure of the O^{-1} concentration.

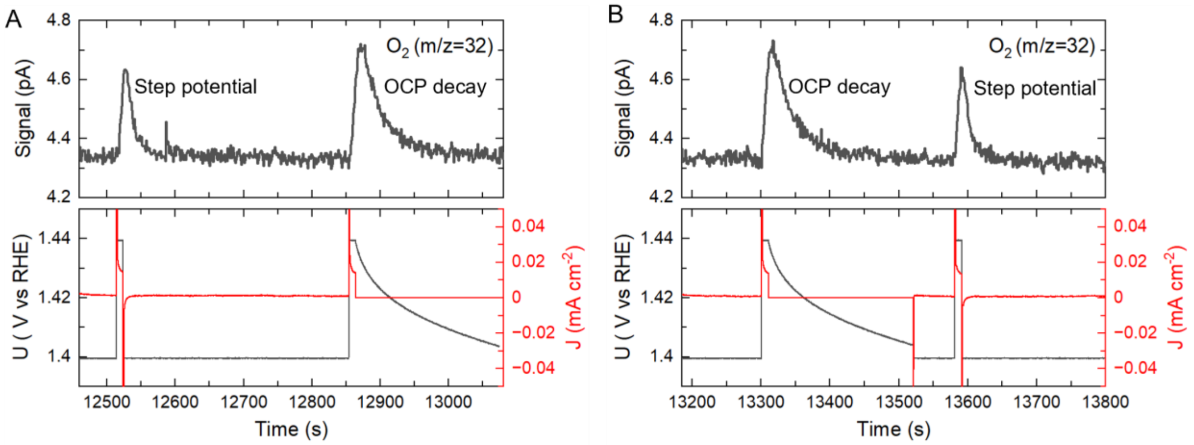

Figure S23 O_2 detection during open circuit potential (OCP) decay. (A) O_2 (m/z = 32) signals detected in EC-MS for step potential measurements from 1.40 to 1.44 V_{RHE} and for a subsequent OCP decay measurement with the same potential steps and holding time. This Figure is also shown is the main text (B) Same measurement but with a reverse order of step potentials and OCP decay measurement.

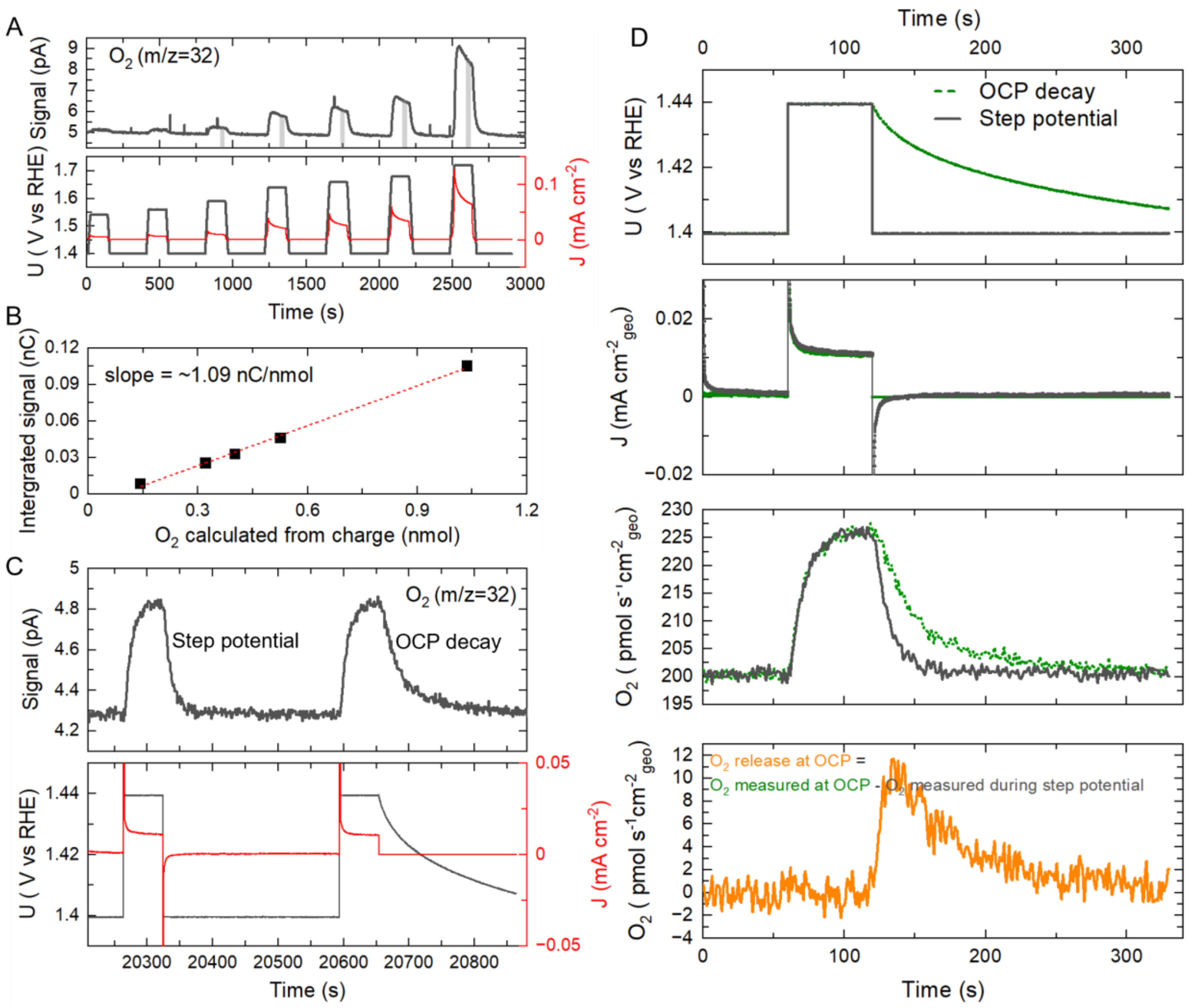

Figure S24 Quantification of O_2 during open circuit potential (OCP) decay. (A) Potential, current and oxygen signal collected in calibration experiment on a clean Pt electrode surface (B) OER calibration curve for Pt electrode. (C) Potential, current and O_2 signal in step potential measurements and OCP decay measurements. (D) Comparison of applied potential during OCP and step potentials (top panel), the corresponding current density (second top panel), calibrated O_2 signal detected (second bottom) and the net rate of O_2 release during OCP decay. The O_2 release at OCP is calculated by the subtraction of calibrated O_2 signal from OCP decay with that from step potential measurement.

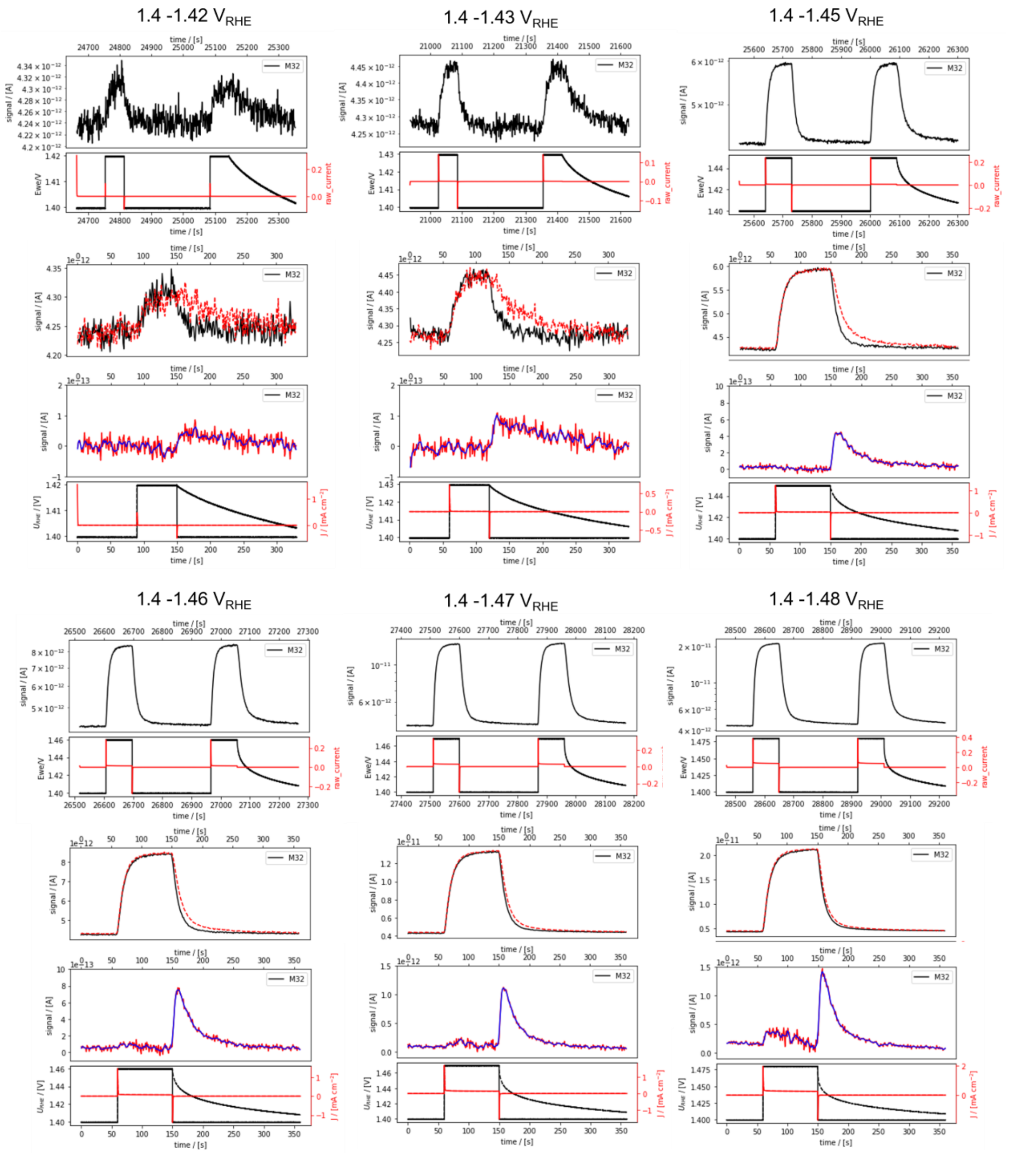

Figure S25 Quantification of O_2 during open circuit potential (OCP) decay-measurement 1. Comparison of applied potential, the corresponding current density (second top panel), calibrated O_2 signal detected (second bottom) and the net rate of O_2 release during OCP decay and step potentials measurements at a series of potentials. The O_2 release at OCP is calculated by the subtraction of calibrated O_2 signal from OCP decay with that from step potential measurement.

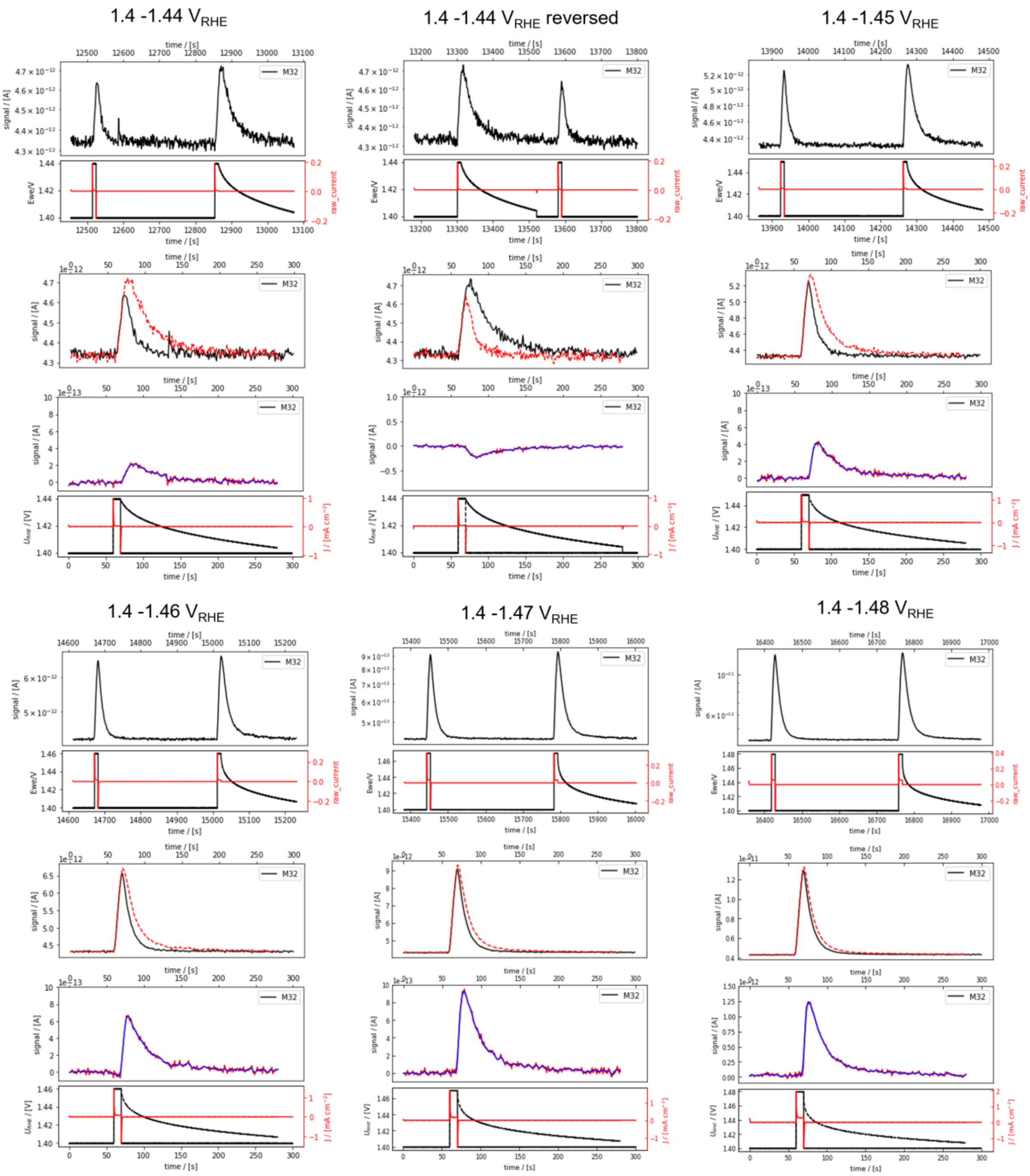

Figure S26 Quantification of O_2 during open circuit potential (OCP) decay-measurement 2. Comparison of applied potential, the corresponding current density (second top panel), calibrated O_2 signal detected (second bottom) and the net rate of O_2 release during OCP decay and step potentials measurements at a series of potentials. The O_2 release at OCP is calculated by the subtraction of calibrated O_2 signal from OCP decay with that from step potential measurement.

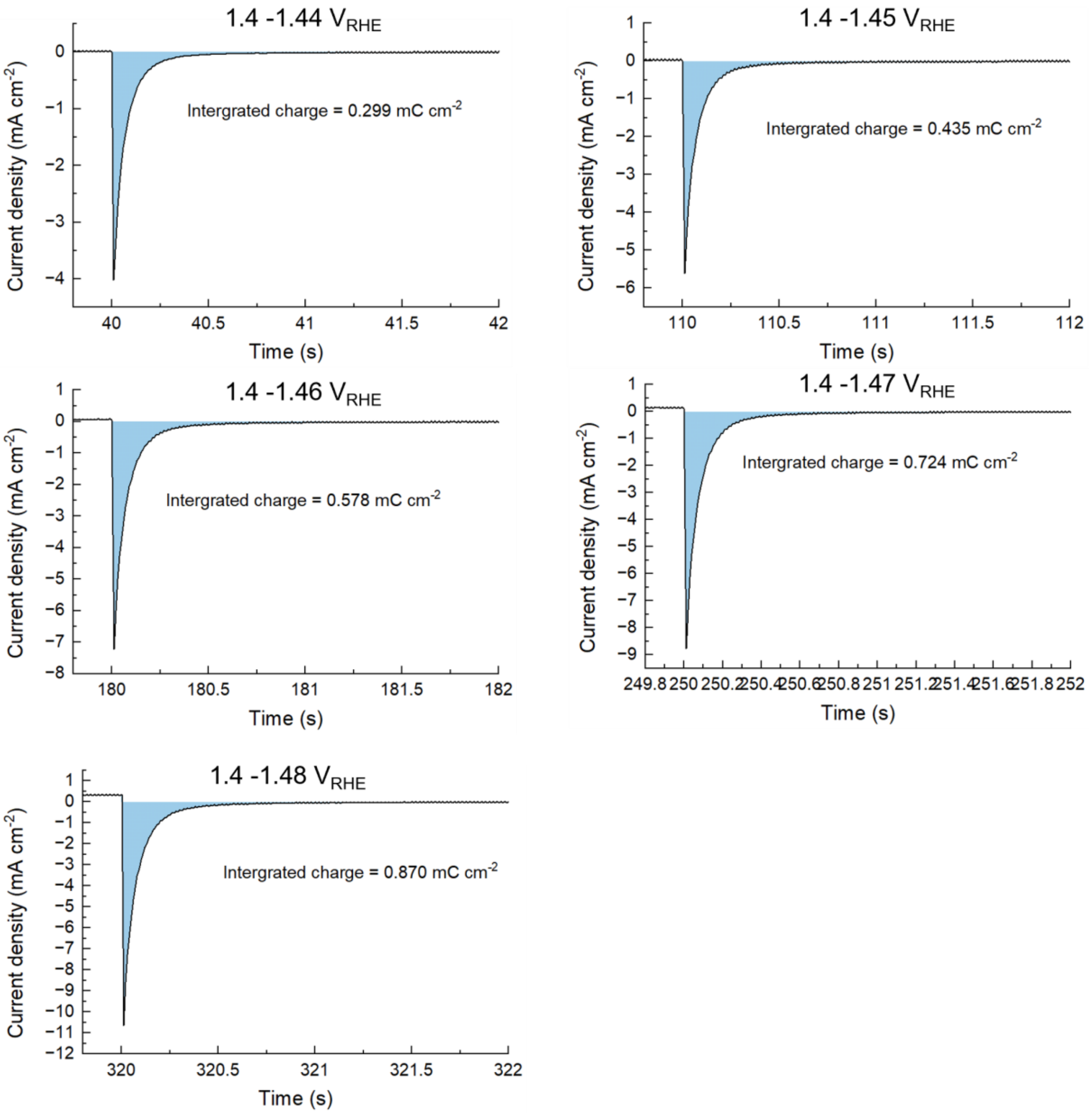

Figure S27 Integration of cathodic current spike during step potential measurements, starting from 1.415 V_{RHE} to various higher potentials and back to 1.415 V_{RHE}. This cathodic peak indicates the charge required to reduce the accumulated O^{1-} back to O^{2-} at 1.415 V_{RHE}, a potential chosen based on the absence of significant oxygen release observed below this threshold during OCP decay.

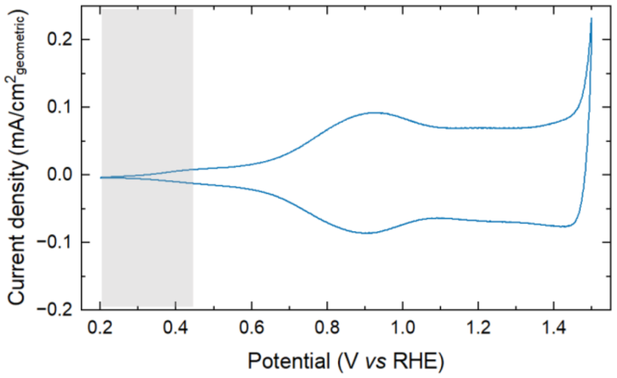

Figure 28 Cyclic voltammetry of IrO_x measured in 0.1 M $HClO_4$ at a scan rate of 10 mV s^{-1}, over the potential range 0.2–1.5 V_{RHE}. The grey-shaded region marks the potential window where minimal redox process and predominantly double-layer charging are expected.

Supplementary Note 8

Iridium dissolution during OCP decay measurements

The OCP decay is intrinsically a discharge process, and in principle a pathway involving Ir dissolution and O_2 evolution could also contribute. To examine this possibility, we specifically measured Ir dissolution during the OCP protocol using Inductively Coupled Plasma Mass Spectrometry (ICP-MS). ICP-MS (Agilent 8900 ICP-QQQ) was employed to quantify the concentration of dissolved Ir following OCP decay, using helium as the collision gas. The instrument was equipped with an Agilent SPS 4 open-bed autosampler, an Ultra High Matrix Introduction (UHMI) system, a quartz spray chamber and torch, and a nickel sampling cone. Samples and standards were analysed in triplicate in single quadrupole mode, with the instrument tuned using a standard tuning solution. Reported values represent the mean of three measurements, with standard deviations below 0.1 ppb. Calibration was performed against Ir standards ($IrCl_3$ in 7% HCl, 1000 mg L^{-1} Ir, Certipur®) diluted in 0.1 M $HClO_4$ to 0, 0.05, 0.5, 1, 5, 10, 50, and 100 ppb. The resulting calibration curve exhibited a coefficient of determination (R^2) greater than 0.999.

Since it is challenging to isolate dissolution originating solely from the OCP decay (excluding contributions from potential ramps or holds), we minimised the holding time to 3 s while keeping the decay time for 90 s, and estimated an upper bound of dissolution during OCP decay by assuming that all of the observed Ir loss originates from the decay. Each OCP decay was repeated 10 times to get an average of the dissolution for a single OCP decay. As shown in Fig. S29, even with this overestimation, the measured Ir loss was only 0.0046-0.0056 nmol cm^{-2} during decay at potentials from 1.44 to 1.48 V_{RHE}, which is ~2-3 orders of magnitude lower than the amount of oxygen released during the decay (0.53-1.75 nmol cm^{-2}). Based on these results, we consider it less likely that oxygen release during OCP decay arises from structural changes involving Ir dissolution.

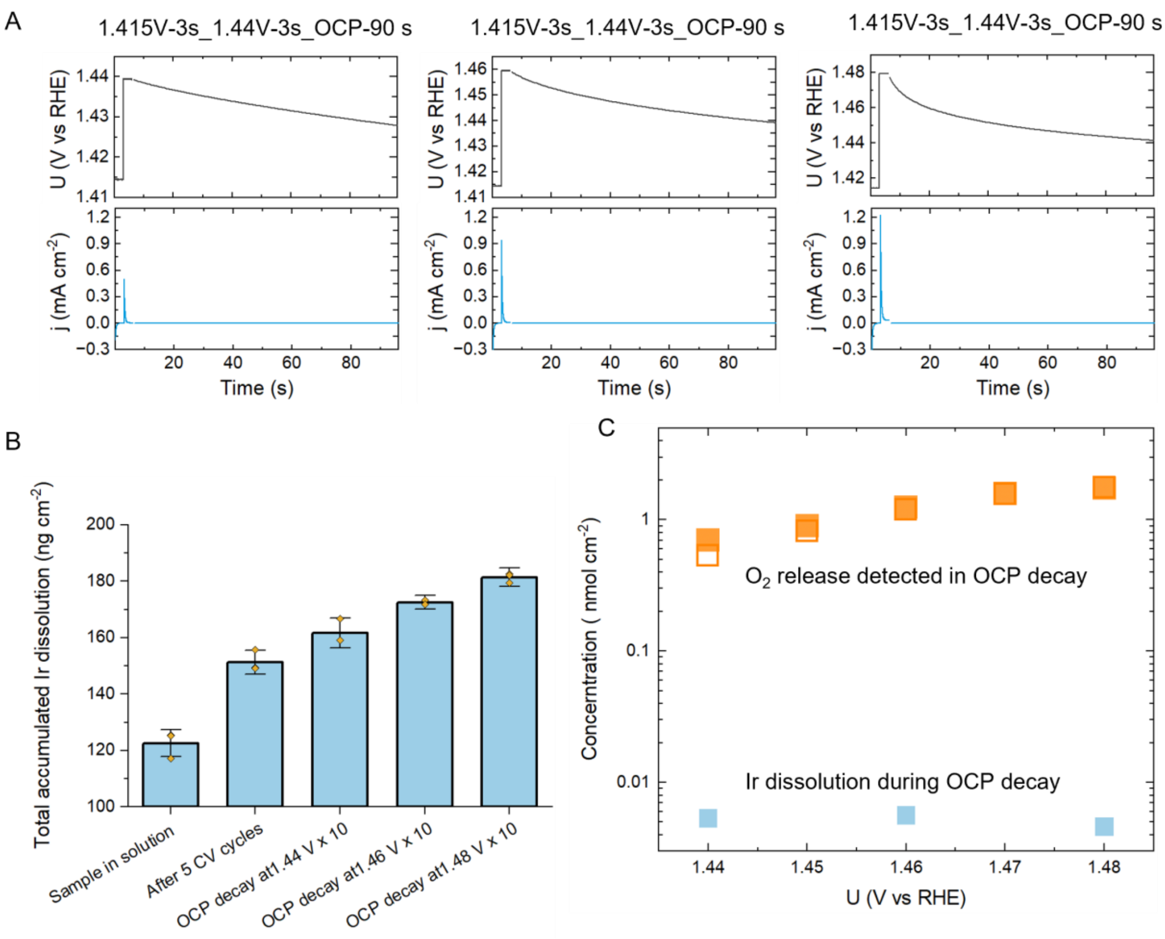

Figure S29 ICP-MS measurements during OCP decay. A) Potential and current profiles during OCP decay protocols at 1.44, 1.46, and 1.48 V_{RHE}. Since it is challenging to isolate dissolution originating solely from the OCP decay (excluding contributions from potential ramps or holds), we minimised the holding time to 3 s while keeping the decay time for 90 s, and estimated an upper bound of dissolution during OCP decay by assuming that all of the observed Ir loss originates from the decay. Each OCP decay was repeated ten times, and the current and potential traces shown correspond to the final repeat. B) Accumulated Ir dissolution in the electrolyte after different measurements. The ICP-MS measurement procedures were carried out sequentially from left to right. Data are presented as mean values +/- standard deviation and overlaid with the corresponding data points of three measurements. C) Comparison between the amount of oxygen released and the Ir dissolved into the electrolyte at different potentials during OCP decay. Oxygen release data are adapted from Figure 4h in the main text. The average difference in Ir concentration between post-CV and post-OCP decay (×10 repeats) at 1.44 V was taken as the dissolution associated with the OCP decay protocol at 1.44 V, while dissolution from OCP decay at 1.46 V and 1.48 V are calculated from the difference between OCP decay at 1.46 V and OCP decay at 1.44 V, and between 1.46 V and 1.48 V, respectively.

Supplementary Note 9

Relation between oxygen evolution rate vs accumulated O^{-1} concentrations

Recent studies, including our own,[2, 3, 14, 15] have discussed kinetic models for water oxidation on metal oxides including iridium oxides and photocatalysts like hematite. We first carried out such an analysis by assuming a typical rate-raw behaviour. We consider O^{-1} to be the active species governing the rate-determining O–O bond formation step, and the other steps to have negligible influence on the overall reaction rate. Mathematically, this can be represented as:

$$r_{oxygen} = k[O^{-1}]^n$$

where r_{oxygen} is the oxygen evolution rate detected in electrochemical mass spectrometry, k is the reaction rate constant, $[O^{-1}]$ is the concentration of O^{-1} accumulated, and n is the reaction order. By plotting the oxygen evolution rate against the concentration of O^{-1} species (i.e., species formed during redox transition three in our optical spectroscopy), we constructed a log–log plot of $\log(r_{oxygen})$ vs $\log([O^{-1}])$ to estimate the reaction order n. As shown in Fig. S30, the log–log plot gives a roughly linear relation; however, the reaction order extracted from the slope is unphysically high ($n \approx 10.6$). This suggests that the assumption of simple rate-law kinetics may not apply here, and that alternative kinetic models should be considered. Recent reports, including ours, [2, 3, 14-16,] indicate that the reaction rate (current density) increases exponentially with hole coverage, as hole accumulation linearly lowers the activation energy for O–O bond formation. To test this with our dataset, we also plotted $\log(r_{oxygen})$ against $[O^{-1}]$ on a linear scale. This plot shows a similarly linear trend, which seems to support the idea that the rate increases approximately exponentially with $[O^{-1}]$. We note that this behaviour is in sharp contrast to photocatalyst oxides such as hematite,[67,68] where well-defined reaction orders of one or three have been reported. Instead, our findings are consistent with recent studies on Iridium oxides showing that the oxygen evolution rate increases exponentially with O^{-1} (or hole) coverage, rather than following a simple rate law.

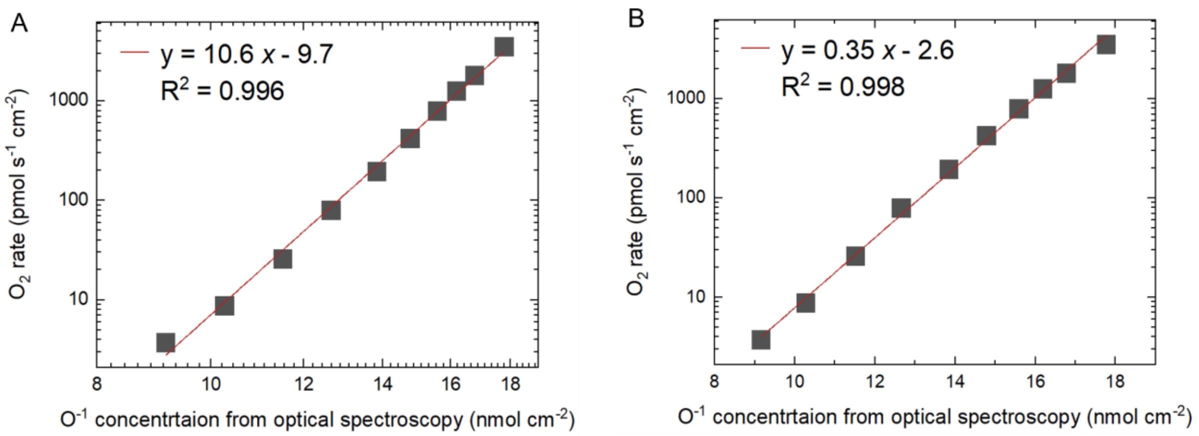

Figure S30. a) Log–log plot examining the rate-law dependence of oxygen release rate on O^{-1} concentration, based on the rate expression $r_{oxygen} = k[O^{-1}]^n$. The oxygen release rate is derived from electrochemical mass spectrometry, and the O^{-1} concentration from optical spectroscopy deconvolution (densities of redox transition 3). b) Semi-log plot examining the exponential dependence of oxygen release rate on O^{-1} concentration, following $r_{oxygen} = k\ exp[(m*[O^{-1}]^n + b)/k_bT]$. The oxygen release rate is derived from electrochemical mass spectrometry, and the O^{-1} concentration from optical spectroscopy deconvolution (density of redox transition 3)

References

(1) Henkelman, G.; Arnaldsson, A.; Jónsson, H. A fast and robust algorithm for Bader decomposition of charge density. *Computational Materials Science* **2006**, *36* (3), 354-360. DOI: https://doi.org/10.1016/j.commatsci.2005.04.010.

(2) Liang, C.; Rao, R. R.; Svane, K. L.; Hadden, J. H. L.; Moss, B.; Scott, S. B.; Sachs, M.; Murawski, J.; Frandsen, A. M.; Riley, D. J.; et al. Unravelling the effects of active site density and energetics on the water oxidation activity of iridium oxides. *Nature Catalysis* **2024**, *7* (7), 763-775. DOI: 10.1038/s41929-024-01168-7.

(3) Liang, C.; Katayama, Y.; Tao, Y.; Morinaga, A.; Moss, B.; Celorrio, V.; Ryan, M.; Stephens, I. E. L.; Durrant, J. R.; Rao, R. R. Role of Electrolyte pH on Water Oxidation for Iridium Oxides. *Journal of the American Chemical Society* **2024**, *146* (13), 8928-8938. DOI: 10.1021/jacs.3c12011.

(4) Padgett, E.; Yu, H.; Blair, S. J.; Cullen, D. A.; Ahluwalia, R. K.; Myers, D. J.; Pivovar, B.; Alia, S. M. Quantifying Sources of Voltage Decay in Long-Term Durability Testing for PEM Water Electrolysis. *Journal of The Electrochemical Society* **2025**, *172* (5), 054508. DOI: 10.1149/1945-7111/add184.

(5) Tsai, H. M.; Babu, P. D.; Pao, C. W.; Chiou, J. W.; Jan, J. C.; Krishna Kumar, K. P.; Chien, F. Z.; Pong, W. F.; Tsai, M.-H.; Chen, C.-H.; et al. Comparison of electronic structures of RuO2 and IrO2 nanorods investigated by x-ray absorption and scanning photoelectron microscopy. *Applied Physics Letters* **2007**, *90* (4). DOI: 10.1063/1.2430929 (acccessed 3/14/2024).

(6) Pfeifer, V.; Jones, T. E.; Velasco Velez, J. J.; Massue, C.; Greiner, M. T.; Arrigo, R.; Teschner, D.; Girgsdies, F.; Scherzer, M.; Allan, J.; et al. The electronic structure of iridium oxide electrodes active in water splitting. *Phys Chem Chem Phys* **2016**, *18* (4), 2292-2296. DOI: 10.1039/c5cp06997a.

(7) Pfeifer, V.; Jones, T. E.; Wrabetz, S.; Massue, C.; Velasco Velez, J. J.; Arrigo, R.; Scherzer, M.; Piccinin, S.; Havecker, M.; Knop-Gericke, A.; et al. Reactive oxygen species in iridium-based OER catalysts. *Chem Sci* **2016**, *7* (11), 6791-6795. DOI: 10.1039/c6sc01860b.
(8) Velasco-Velez, J. J.; Pfeifer, V.; Hävecker, M.; Weatherup, R. S.; Arrigo, R.; Chuang, C. H.; Stotz, E.; Weinberg, G.; Salmeron, M.; Schlögl, R.; et al. Photoelectron Spectroscopy at the Graphene–Liquid Interface Reveals the Electronic Structure of an Electrodeposited Cobalt/Graphene Electrocatalyst. *Angewandte Chemie International Edition* **2015**, *54* (48), 14554-14558. DOI: 10.1002/anie.201506044.
(9) Kumar, S.; Counter, J. J. C.; Grinter, D. C.; Spronsen, M. A. V.; Ferrer, P.; Large, A.; Orzech, M. W.; Jerzy Wojcik, P.; Held, G. An electrochemical flow cell for operando XPS and NEXAFS investigation of solid–liquid interfaces. *Journal of Physics: Energy* **2024**, *6* (3), 036001. DOI: 10.1088/2515-7655/ad54ee.
(10) Pasquini, C.; Liu, S.; Chernev, P.; Gonzalez-Flores, D.; Mohammadi, M. R.; Kubella, P.; Jiang, S.; Loos, S.; Klingan, K.; Sikolenko, V.; et al. Operando tracking of oxidation-state changes by coupling electrochemistry with time-resolved X-ray absorption spectroscopy demonstrated for water oxidation by a cobalt-based catalyst film. *Analytical and Bioanalytical Chemistry* **2021**, *413* (21), 5395-5408. DOI: 10.1007/s00216-021-03515-0.
(11) Trimarco, D. B.; Scott, S. B.; Thilsted, A. H.; Pan, J. Y.; Pedersen, T.; Hansen, O.; Chorkendorff, I.; Vesborg, P. C. K. Enabling real-time detection of electrochemical desorption phenomena with sub-monolayer sensitivity. *Electrochimica Acta* **2018**, *268*, 520-530. DOI: https://doi.org/10.1016/j.electacta.2018.02.060.
(12) Scott, S. B.; Rao, R. R.; Moon, C.; Sørensen, J. E.; Kibsgaard, J.; Shao-Horn, Y.; Chorkendorff, I. The low overpotential regime of acidic water oxidation part I: the importance of O2 detection. *Energy & Environmental Science* **2022**, *15* (5), 1977-1987, 10.1039/D1EE03914H. DOI: 10.1039/D1EE03914H.
(13) Cherevko, S.; Zeradjanin, A. R.; Topalov, A. A.; Kulyk, N.; Katsounaros, I.; Mayrhofer, K. J. J. Dissolution of Noble Metals during Oxygen Evolution in Acidic Media. *ChemCatChem* **2014**, *6* (8), 2219-2223. DOI: 10.1002/cctc.201402194.
(14) Nong, H. N.; Falling, L. J.; Bergmann, A.; Klingenhof, M.; Tran, H. P.; Spori, C.; Mom, R.; Timoshenko, J.; Zichittella, G.; Knop-Gericke, A.; et al. Key role of chemistry versus bias in electrocatalytic oxygen evolution. *Nature* **2020**, *587* (7834), 408-413. DOI: 10.1038/s41586-020-2908-2.
(15) Risch, M.; Ringleb, F.; Kohlhoff, M.; Bogdanoff, P.; Chernev, P.; Zaharieva, I.; Dau, H. Water oxidation by amorphous cobalt-based oxides: in situ tracking of redox transitions and mode of catalysis. *Energy & Environmental Science* **2015**, *8* (2), 661-674, 10.1039/C4EE03004D. DOI: 10.1039/C4EE03004D.
(16) Righi, G.; Plescher, J.; Schmidt, F.-P.; Campen, R. K.; Fabris, S.; Knop-Gericke, A.; Schlögl, R.; Jones, T. E.; Teschner, D.; Piccinin, S. On the origin of multihole oxygen evolution in haematite photoanodes. *Nature Catalysis* **2022**, *5* (10), 888-899. DOI: 10.1038/s41929-022-00845-9.
